# *GltS* regulates biofilm formation in methicillin-resistant *Staphylococcus aureus*

**DOI:** 10.1038/s42003-022-04239-2

**Published:** 2022-11-23

**Authors:** Miho Shibamura-Fujiogi, Xiaogang Wang, Wiriya Maisat, Sophia Koutsogiannaki, Yunan Li, Yue Chen, Jean C. Lee, Koichi Yuki

**Affiliations:** 1 Department of Anesthesiology, Critical Care and Pain Medicine, Cardiac Anesthesia Division, Boston Children’s Hospital, Boston, MA USA; 2 Department of Anaesthesia and Immunology, Harvard Medical School, Boston, MA USA; 3 Department of Medicine, Brigham and Women’s Hospital and Harvard Medical School, Boston, MA USA; 4 Department of Biochemistry, Molecular Biology and Biophysics, University of Minnesota, Minneapolis, MN USA

**Keywords:** Bacterial infection, Biofilms

## Abstract

Biofilm-based infection is a major healthcare burden. Methicillin-resistant *Staphylococcus aureus* (MRSA) is one of major organisms responsible for biofilm infection. Although biofilm is induced by a number of environmental signals, the molecule responsible for environmental sensing is not well delineated. Here we examined the role of ion transporters in biofilm formation and found that the sodium-glutamate transporter *gltS* played an important role in biofilm formation in MRSA. This was shown by *gltS* transposon mutant as well as its complementation. The lack of exogenous glutamate also enhanced biofilm formation in JE2 strain. The deficiency of exogenous glutamate intake accelerated endogenous glutamate/glutamine production, which led to the activation of the urea cycle. We also showed that urea cycle activation was critical for biofilm formation. In conclusion, we showed that *gltS* was a critical regulator of biofilm formation by controlling the intake of exogenous glutamate. An intervention to target glutamate intake may be a potential useful approach against biofilm.

## Abstract (web summary)

Impaired intake of exogenous glutamate, due to deficiency of the GltS sodium-glutamate transporter, promotes increased Staphylococcus aureus biofilm formation in vitro and in vivo.

## Introduction

Biofilms are aggregates of bacteria that are adherent to a material surface and encased in a self-synthetic extracellular polymer matrix. Biofilm-related infections occupy a large percentage of nosocomial infections^1^, including device and indwelling catheter infections. Biofilm infections are highly resistant to antibiotic therapy and host defense, making biofilms difficult to be eradicated^2^. The transition of bacterial cells from planktonic to biofilm form is induced by the regulation of gene expression in response to sensed physicochemical environmental signals such as ions, pH, and nutrients, which in turn affects various signaling pathways^3^. Methicillin-resistant *Staphylococcus aureus* (MRSA) is a leading nosocomial pathogen and commonly associated with significant morbidity, hospital mortality, length of stay, and economic burden, with an estimated attributable healthcare cost of $1.7 billion in 2017 in the United States^4^. An investigation into the underlying molecular mechanisms of biofilm formation in MRSA is critical to developing a therapeutic approach.

The tricarboxylic acid (TCA) cycle is a central metabolic pathway that provides energy, reducing potential, and biosynthetic intermediates. During biofilm formation, the TCA cycle may be initially activated^5^, but the inactivation of the TCA cycle generally ensues^6^, contributing to an increase in the percentage of protein components in the USA300 biofilm matrix^7,8^. The involvement of the urea cycle in biofilm formation is also described^7^. Ion transporters play a critical role in sensing environments and regulating ion trafficking. We have previously shown that the a subset of ion transporters was involved in biofilm formation in *Escherichia coli*^9^. The biological roles of ion transporters in MRSA were recently studied by several investigators^10–13^. For example, *alsT* functions as an efficient glutamine transporter^13^ and *gltT* is an aspartate transporter^11^. However, their role in biofilm has not been extensively studied. With the hypothesis that a subset of MRSA ion transporters would be involved in biofilm formation, we screened biofilm formation for ion transposon (Tn) mutants in the Nebraska library^14^. We found significantly increased biofilm formation in a major sodium-glutamate symporter *gltS* transposon mutant strain (*gltS::Tn*)^13^ compared to the parental strain. Because the *gltS* mutant was the only one that demonstrated alteration in biofilm formation among all the sodium ion transporters, we tested our hypothesis that glutamate-induced metabolic changes would affect MRSA biofilm formation by utilizing chemically defined media (CDM) depleted of glutamate, mass spectrometry-based metabolomic analyses, and RNA transcriptomic analyses. Although the involvement of the TCA and urea cycles in biofilm formation has been previously described as above^7,15,16^, it is not known how exogenous glutamate, a major amino acid in central metabolism, is involved in biofilm formation in conjunction with the TCA and urea cycles. Understanding the role of glutamate uptake via *gltS* in MRSA biofilm formation will provide an insight into mechanisms that regulate biofilm formation.

## Results

### *GltS* transposon mutant enhanced biofilm formation in vitro

**Fig. 1:**
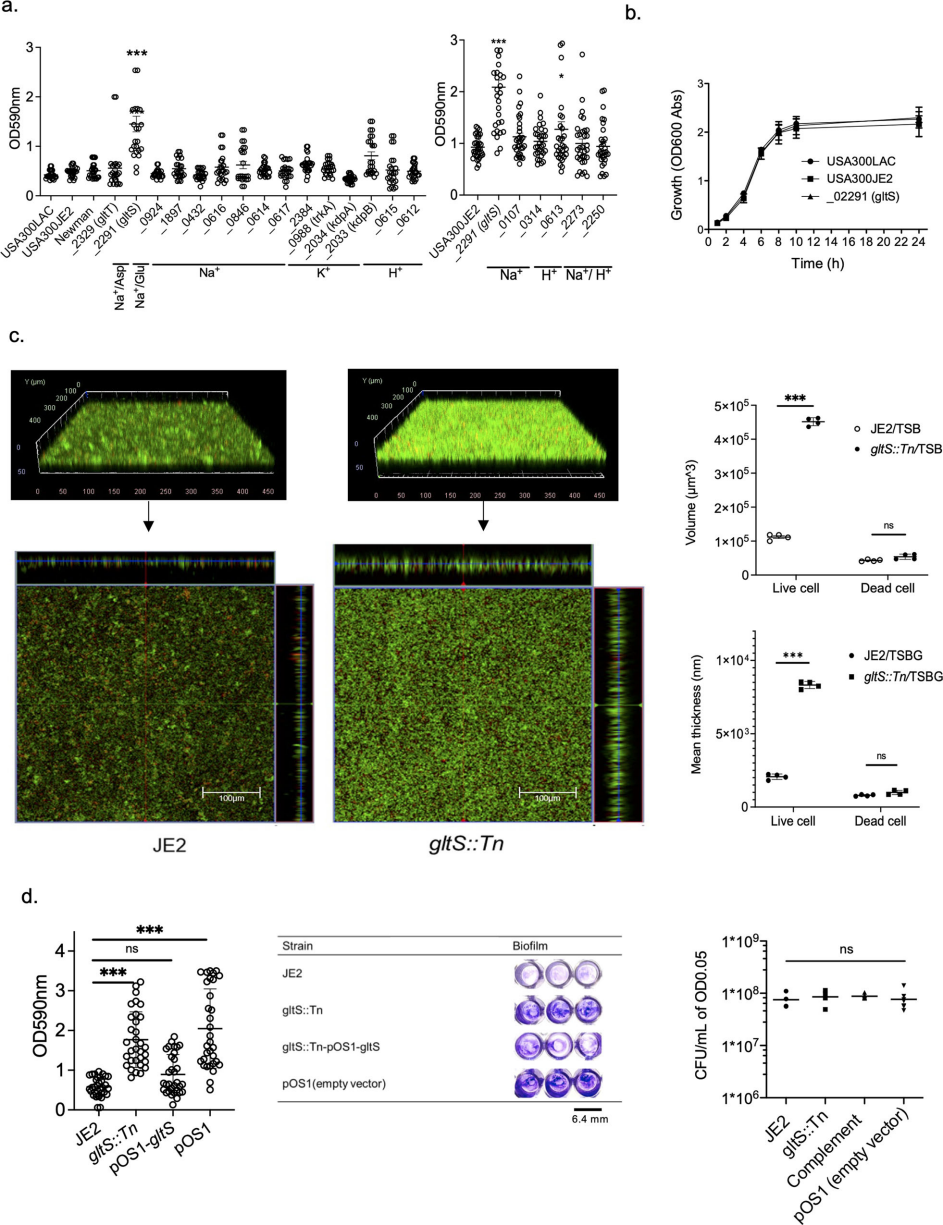
Biofilm formation of *gltS* transposon mutant in vitro. **a** Crystal-violet based biofilm screening of ion transporter mutants. Biofilm quantification in 1% glucose TSB medium at 48 h. We included accession number for all the genes. For one that have specific gene name, we included it in parenthesis. For example, *gltT* has gene accession number of SAUSA300_2329. In this case, we show as _*2329* (*gltT*). If we do not have gene number such as gene with accession number of SAUSA300_0924, we just show accession number as _0924. Data are shown as mean ± SD of three independent tests with 8 replicates. One-way ANOVA with *post hoc* Bonferroni’s multiple comparisons test vs USA300 JE2. ***p < 0.001, *p < 0.05. **b** Growth curves of USA300 parental strain, and *gltS* mutant strain by OD600 absorption value of turbidity. **c** (Left) Orthogonal, z-stacked images of biofilms with a confocal laser microscope. The 48-h biofilm was subjected to live/dead staining. Live cells are shown in green (488 nm excitation laser) and dead cells are shown in red (570 nm wavelength filter). A representative image of two independent experiments is shown. The volume of biofilm calculated by using the voxel counter plugin in Image J and shown as mean ± SD of four independent locations. One-way ANOVA with *post hoc* Bonferroni’s multiple comparisons test vs USA300 JE2. ***p < 0.001. **d** Crystal violet-based biofilm assay using complemented Δ*gltS* strain (*gltS::Tn*-pOS1-*gltS*) at 48 h. One-way ANOVA with *post hoc* Bonferroni analysis. ***p < 0.001. (Middle) OD measurement of eight replicates is shown as mean ± SD. Crystal violet-stained Images. (Right) Because we used solution with O.D. of 0.05 as an input for biofilm assay, we compared CFU with O.D. of 0.05 for each strain. Data is shown as mean ± SD of 6 replicates. Statistical significance was tested using one-way ANOVA with *post hoc* Bonferroni analysis. No difference was observed.

To test the hypothesis that a subset of ion transporters would be responsible for biofilm formation, we first performed screening experiments using a 96-well format crystal violet-based biofilm assay. The list of Nebraska library strains that we tested is shown in Supplementary Data 1, and the results are shown in Fig. 1a. Among the 20 transposon mutants tested, *gltS* transposon mutant (*gltS::Tn*) significantly enhanced biofilm formation compared to the parent strain JE2. Potassium-transporting ATPase ATP-binding protein mutant (*kdpB::Tn*) also showed enhanced biofilm formation compared to that of the parent strain, although less than *gltS::Tn* (Fig. 1a). The growth was similar for the parent and *gltS::Tn* strains (Fig. 1b), indicating that bacterial growth rates did not account for the phenotype. We also examined the growth of all the other mutants we tested in Fig. 1a. No mutants showed any significant difference in growth compared to their parent strain (Supplementary Data 2). We examined biofilm mass by assessing live/dead cell-stained samples in two-well plastic chamber slides. The z-stack images of the biofilm at 2.45 μm intervals showed cells deposited in the biofilm at a depth of approximately 22.05 μm for the parental strain and 36.75 μm for the *gltS::Tn* strain. (Fig. 1c). The volume of biofilm calculated by using the voxel counter per same surface area for *gltS::Tn* was significantly larger than the one of JE2 (Fig. 1c), consistent with the results of the crystal violet-based assay (Fig. 1a). The thickness data also corresponded to the volume (Fig. 1c). The thickness of live cells in *gltS::Tn* was significantly larger than in JE2. To confirm that the increased biofilm formation of the *gltS::Tn* mutant was due to the *gltS* deficiency and not due to any secondary mutations in the genome, the complementation of *gltS* was performed. The biofilm formation of the complemented strain was comparable to that of JE2, indicating that *gltS* deficiency was responsible for biofilm enhancement (Fig. 1d).

To evaluate the differences in biofilm formation and components among the strains, we compared the expression levels of genes involved in biofilm component and performed biofilm assays using each reagent treatment on biofilm supernatants. The biofilms of the parental strain and *gltS::Tn* were reduced to a similar degree by proteinase K treatment (Supplementary Fig. 1a, 56.8%, and 66.8% reduction, respectively). There was a significant reduction in the amount of *gltS::Tn* biofilm after DNase treatment at 24 h, not in the parent strain (Supplementary Fig. 1b). At 48 h, DNase treatment attenuated *gltS::Tn* biofilm amount, although not statistically significant. Not all eDNA could be digested by the DNase I test, since several types of DNA-binding proteins in *S. aureus*, such as phenol soluble modulin (PSM) peptides and Eap, can protect eDNA from digestion by DNase I^17^. Although we did not find any difference in the percentage of protein among biofilm constituents, the type of proteins is important in biofilm formation.

### *GltS::Tn* mutant enhanced biofilm formation in vivo

**Fig. 2:**
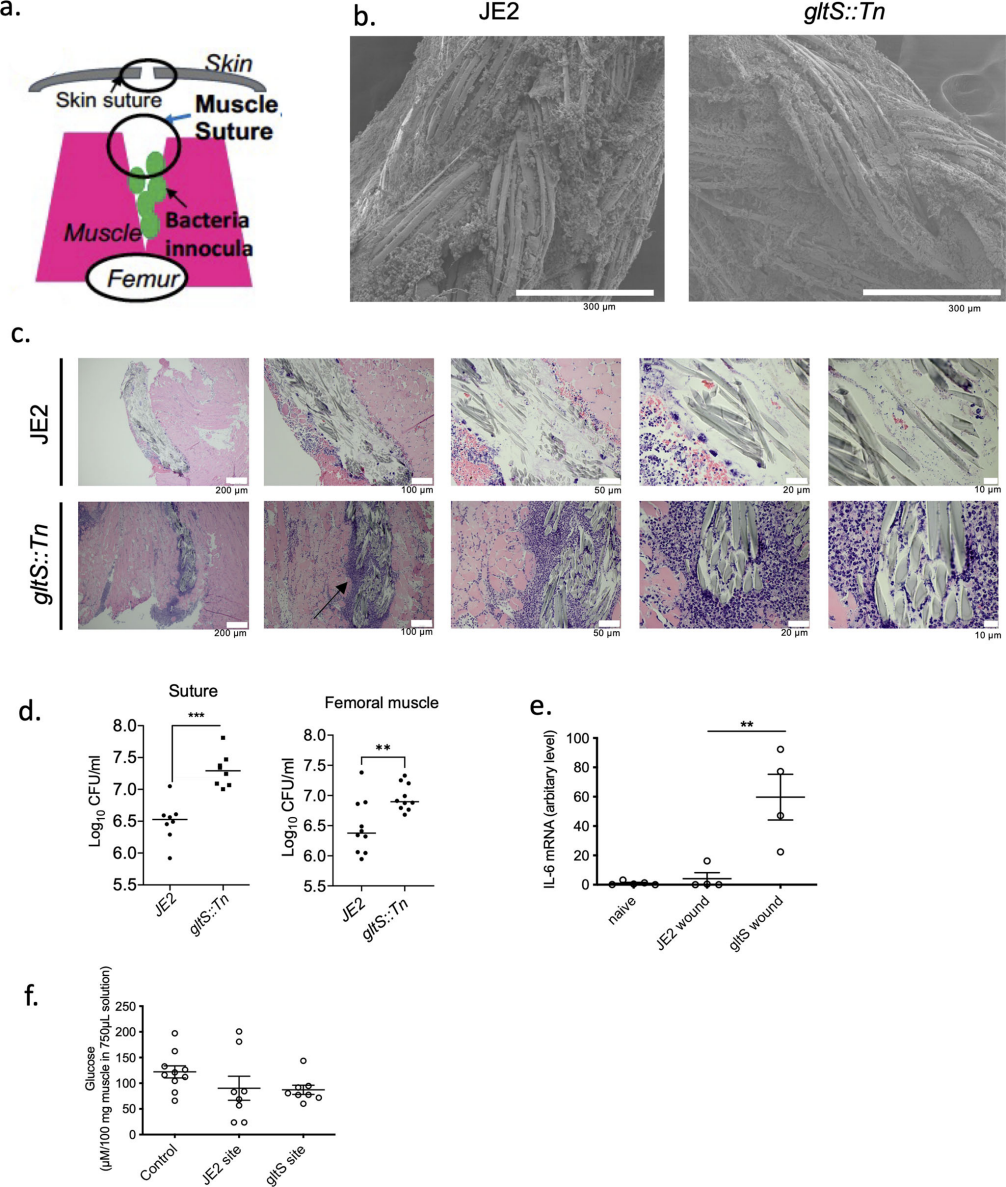
Biofilm formation of *gltS* transposon mutant in vivo. **a** Scheme of a murine wound infection model. **b** SEM image of muscle sutures. Representative images from two independent experiments. Scale bar (white) in each image. **c** Hematoxylin and eosin (H&E) staining of the surgical infection site. Scale bar (white) in each image. A black arrow indicates extensive leukocyte infiltration. **d** Sutures were sonicated in the same volume of PBS for each strain, and the CFUs were counted. Muscle sections of the same weight from each mouse were homogenized, and the CFUs in the homogenized suspensions were quantified. Data are shown as mean ± SD of 8 mice for suture and 10 mice for muscle sample. **e** IL-6 mRNA levels in the wound tissues at 48 h after infection with JE2 or *gltS::Tn* were examined using RT-qPCR. Data are shown as mean ± SD of 4 replicates. One way ANOVA with *post hoc* Bonferroni analysis was done. **p < 0.01. **f** Glucose levels at the wound were measured. Data are shown as mean ± SD of 6 replicates. One way ANOVA with post hoc Bonferroni analysis was done. No statistical significance was observed.

To examine the role of *gltS* in vivo, we sought for an in vivo biofilm formation model. Deep muscle infection model is a model created by inoculating MRSA, followed by muscle suture closure as well as skin closure (Fig. 2a). In both JE2 and *gltS::Tn* strains, the biofilm formation was seen on the muscle sutures by electron microscopy^18^ (Fig. 2b). Histology analysis demonstrated more extensive leukocyte infiltration following *gltS::Tn* infection (Fig. 2c). Bacterial loads of both muscle suture and muscles showed that *gltS::Tn* infection was associated with a significantly greater bacterial burden (Fig. 2d), in line with our in vitro data. IL-6 is one of the indicators for organ injury. IL-6 level was higher in *gltS::Tn* treated wound (Fig. 2e), suggesting more tissue damage from *gltS::Tn* infection. Lastly we examined glucose level in the wound. Glucose level at the wound site infected with JE2 or *gltS::Tn* showed a tendency to be lower than the baseline, but the difference was not statistically significant (Fig. 2f).

### Exogeneous glutamate deficiency enhanced biofilm formation

**Fig. 3:**
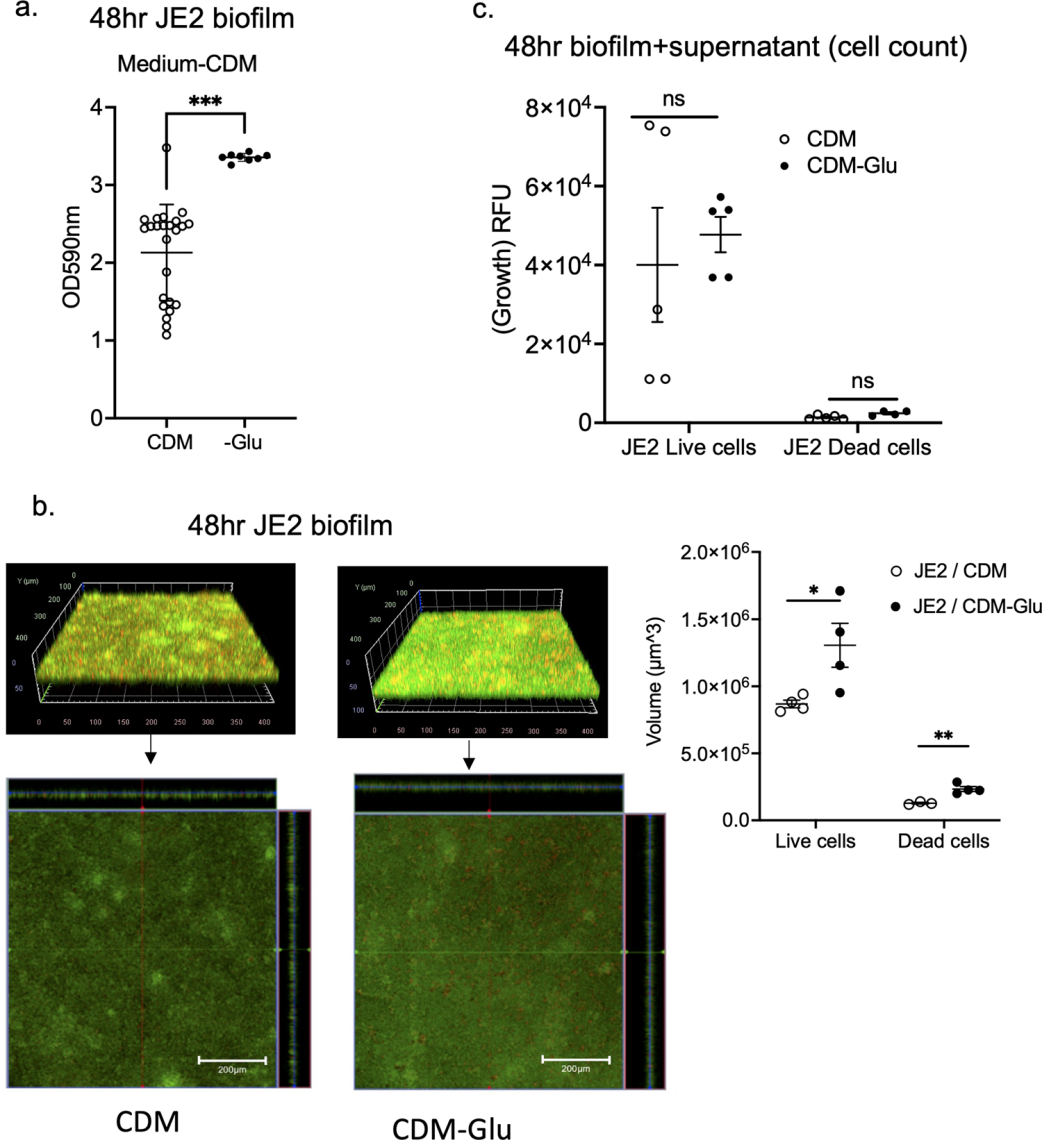
The role of exogenous glutamate in biofilm formation. **a** Absorbance values of crystal violet assays of 48 h JE2 biofilms using complete CDM containing glucose medium (CDM) and glutamate (Glu)-depleted from CDM medium (CDM-Glu). Data are shown as mean ± SD of 8 replicates. Student t test was performed. ***p < 0.001. **b** Images of JE2 biofilm after LIVE/DEAD staining by CLSM and channel-specific volume calculated by Image J. The volume of biofilm calculated by using the voxel counter plugin in Image J and shown as mean ± SD of four independent locations. One-way ANOVA with *post hoc* Bonferroni’s multiple comparisons test vs USA300 JE2. * and **p < 0.05 and *p* < 0.01. **c** Cell counts were determined by using fluorescence intensity of the whole 48-h biofilm and supernatant as a surrogate. Fluorescence intensity was measured as relative fluorescence unit (RFU) because the spectra of collected light should be corrected for instrument effects. Data are shown as mean ± SD of 8 replicates. One-way ANOVA with *post hoc* Bonferroni’s multiple comparisons test vs USA300 JE2. No statistical significance was observed.

*GltS* is a symporter of Na/glutamate from the environment to the intracellular space. Because *gltS::Tn* mutant was the only one that showed increased biofilm formation among all the Na transporters tested, we hypothesized that exogenous glutamate intake plays a significant role in biofilm formation. A previous study showed that *gltS::Tn* impaired glutamate intake, while *gltT::Tn* (strain 2329) did not show any reduction in glutamate transport^13^. Accordingly, we examined the role of exogenous glutamate in biofilm formation of JE2 parent strain using a chemically defined medium + 1% glucose (CDM) and CDM depleted of glutamate. Glutamate depletion led to a significant increase in biofilm formation assessed by both the crystal violet method (Fig. 3a) and confocal microscopy (Fig. 3b), in line with the *gltS::Tn* phenotype. We measured the number of viable biofilm and planktonic cells using live/death staining using fluorescence intensity as a surrogate. There was no significant difference in cell number between complete CDM and glutamate-depleted medium (Fig. 3c). The results suggested that exogenous glutamate was not essential for cell viability under 48-h culture conditions.

### Intermediate metabolites of the urea cycle and TCA cycle were more abundant in the *gltS::Tn* mutant

**Fig. 4:**
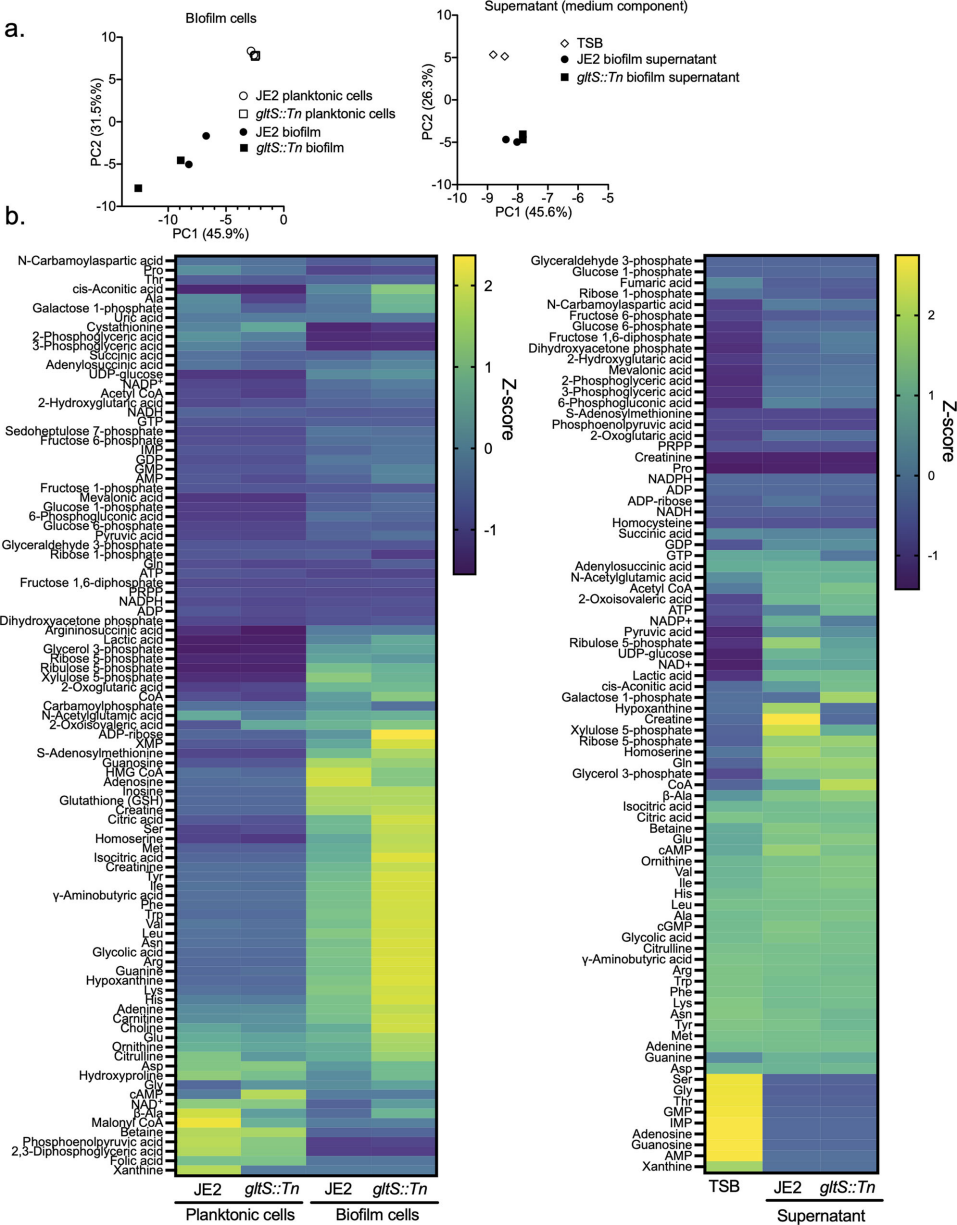
Metabolome profiles of planktonic and biofilm cells and culture supernatants. **a** Principal component analysis (PCA) of metabolites in biofilm cells (left) and supernatant, showing the top two on the x-axis and y-axis, respectively. For supernatant analysis, TSB indicates TSB containing 1% glucose without any bacteria co-culture. **b** Heatmap representation of metabolome profiles analyzed by hierarchical clustering.

Because our data so far showed an inverse correlation between glutamate intake and biofilm formation, we examined the metabolic derangement induced by *gltS::Tn* using a metabolomics analysis. All data in Fig. 4 were from metabolite analysis in biofilm and supernatant using TSB as medium. Figure 4a shows the principal component (PC) analysis; the total contribution of PC1 and PC2 was relatively high, 77.4% in bacterial cells and 71.9% in supernatant. For cells, mevalonic acid, glucose-1-phosphate, and lysine were major contributors to PC1, while succinic acid, β-alanine, and ornithine were major contributors for PC2. On PCA analysis, the supernatant profiles of JE2 and *gltS::Tn* biofilm conditions almost overlapped with each other. Planktonic and biofilm cells showed a clear difference, while a separation of the intracellular metabolite profiles between JE2 and *gltS::Tn* biofilm was limited.

The results of all the measured metabolite profiles were shown by a heatmap (Fig. 4b). Details of pathways will be discussed later; Fig. 4b mainly showed that biofilms in TSB produced a majority of the amino acids, including glutamate, glutamine, and arginine, about 1.4- to 2.0-fold more in *gltS::Tn* than in the parent strain. The absolute concentrations of all metabolites measured are shown in Supplementary Data 3, which includes data from using CDM and glutamate-depleted CDM as medium.

Since parameters such as energy charge, lactate-to-pyruvate ratio, glutathione redox ratio, and total amino acids are useful for gaining further insights into physiological states such as energy and redox status, we listed equations to calculate each metabolic parameter, its relevance to cell metabolism and physiological states, and results in Supplementary Data 4. NAD^+^/NADH ratio indicates metabolic activity^19^. The NAD^+^/NADH ratio of *gltS::Tn*/JE2 strains was 1.6, indicating higher metabolic activity in *gltS::Tn*. This is because NAD^+^, a cofactor in many reactions such as glycolysis, the TCA cycle, and beta-oxidation of fatty acids, was 1.6 times more abundant in *gltS::Tn* biofilm cells than in the parental strain (Supplementary Data 3). The glycerol 3-phosphate/dihydroxyacetone phosphate (DHAP) ratio serves as an indirect parameter for an inverse ratio of NAD^+^/NADH and energy status. The relative ratio of *gltS::Tn*/JE2 strains was low (0.5).

The analyzed metabolites were subjected to metabolic pathway analysis using MetaboAnalyst 5.0 online (Supplementary Data 5). Metabolites with an absolute difference of more than 0.5 in the relative peak area used for metabolome profiling between planktonic cells and 48-h biofilms were included in the analysis. This led to the creation of a heatmap showing metabolites grouped by pathway, focusing on purine metabolism, arginine biosynthesis, pentose phosphate (PP) pathway, and citrate cycle besides the glutamate metabolism (Supplementary Fig. 2a, b). Metabolites generated in the TCA cycle were overall produced in greater abundance in the biofilm state with the rank order of *gltS::Tn* > JE2 strain (Supplementary Fig. 2a). Regarding 2-oxoglutarate (2-OG), there was almost no difference between strains, while glutamate/2-OG ratio was 1.3 times more in *gltS::Tn* compared to JE2. Coenzyme A (CoA) was seen more in *gltS::Tn* cells and supernatant. Similarly, urea cycle related metabolites such as ornithine, citrulline, argininosuccinic acid and arginine were higher in *gltS::Tn* biofilm cells (Supplementary Fig. 2b). Creatine was higher in the supernatant of JE2 strain compared to *gltS::Tn*. As for the PP pathway/glycolysis, the amount of metabolites produced from the planktonic cells relative to the biofilm was higher under PP pathway than under glycolysis (Supplementary Fig. 3a). AMP, hypoxanthine, adenine, XMP, and guanine in purine pathway were produced in higher amounts in *gltS::Tn* (Supplementary Fig. 3b, Supplementary Data 5), in line with higher levels of glutamate and glutamine in the mutant.

### The expression of urea cycle-mediated genes was significantly upregulated in *gltS::Tn*

**Fig. 5:**
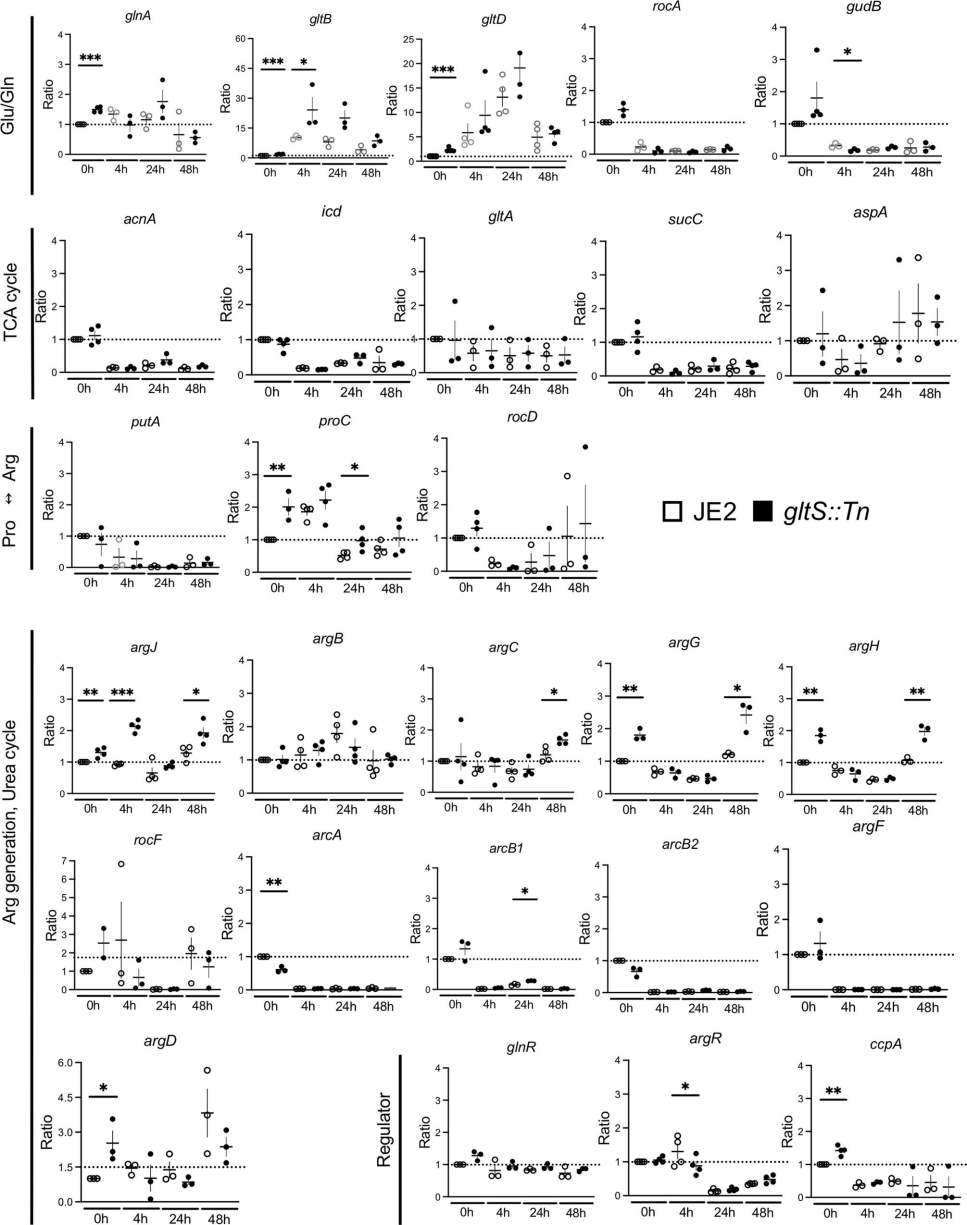
Relative expression of genes related to metabolism and biofilm formation in *S. aureus* as determined by RT-PCR. Expression ratio based on the 0-h value of the JE2 strain over three independent tests. 0 h corresponds to planktonic cells, while 4 h, 24 h and 48 h correspond to biofilm cells at 4, 24, and 48 h-incubation, respectively. Data are mean ± SD of 3–4 replicates. Student t-test between strains of samples collected at the same time. *P < 0.05, **P < 0.01, ***P < 0.001.

Based on the metabolites data described above, we focused on analyzing the transcriptomic profiles of genes involving in the TCA and urea cycles. The genes of enzymes involved in the TCA cycle (*acnA*, *icd*, and *sucC*) were universally downregulated in biofilm cells compared to planktonic cells in both strains (Fig. 5). Although the levels of TCA cycle-mediated metabolites were higher in biofilm cells than in planktonic cells (Supplementary Fig. 2a), RT-qPCR result indicated the presence of inhibitory mechanism for TCA cycle activity. Although TCA cycle-related metabolite levels were higher in *gltS::Tn* mutant compared to the parent strain, there was no significant difference in mRNA expression of the tested genes between the strains. We performed mass spectrometry analysis of JE2 and *gltS::Tn* planktonic and biofilm cells, and compared their TCA cycle related enzyme levels. Some of the TCA cycle related enzymes in *gltS::Tn* cells had higher levels at 24 h. The enzyme levels in *gltS::Tn* cells were consistently higher than those in JE2 strain at 48 h (Supplementary Data 6), consistent with the metabolomics data (Fig. 4). *gltB* and *gltD*, which encode the subunits of glutamine oxoglutarate aminotransferase (GOGAT) and are involved in glutamate and glutamine metabolism, were expressed significantly higher in *gltS::Tn* than in the parental strain at 0 and/or 4 h (Fig. 5), which explained higher glutamate and glutamine levels in *gltS::Tn* biofilm cells. This enzyme was not consistently detected in our mass spectrometry data set.

Glutamate can be shuttled and available for urea cycle metabolism. Our data also showed gene expression of arginine producing molecules were overall upregulated in *gltS::Tn*. An upregulated expression of *argJ* (bifunctional glutamate N-acetyltransferase/amino-acid acetyltransferase) and *argCD* in *gltS::Tn* suggests that glutamate to arginine influx significantly increased, consistent with the result of the metabolic pathway data (Supplementary Fig. 2b). The expression of *argR*, an arginine repressor, was significantly lower in *gltS::Tn* than in the parental strain in 4 h-biofilm cells. These data further demonstrated more active use of the urea cycle in *gltS::Tn* cells.

### RNA sequencing analysis for additional pathway consideration

We applied RNA sequencing experiments (RNA seq) to analyze the expression of genes in pathways other than those examined by RT-qPCR, as well as to understand the expression of supplementary gene subsets associated with the pathways of the focus (Supplementary Fig. 4). Genes that were at least 2-fold more (or less) expressed by RNAseq analysis in *gltS::Tn* strain relative to the parental strain at the time of each cell harvest are shown in Supplementary Data 7.

**Fig. 6:**
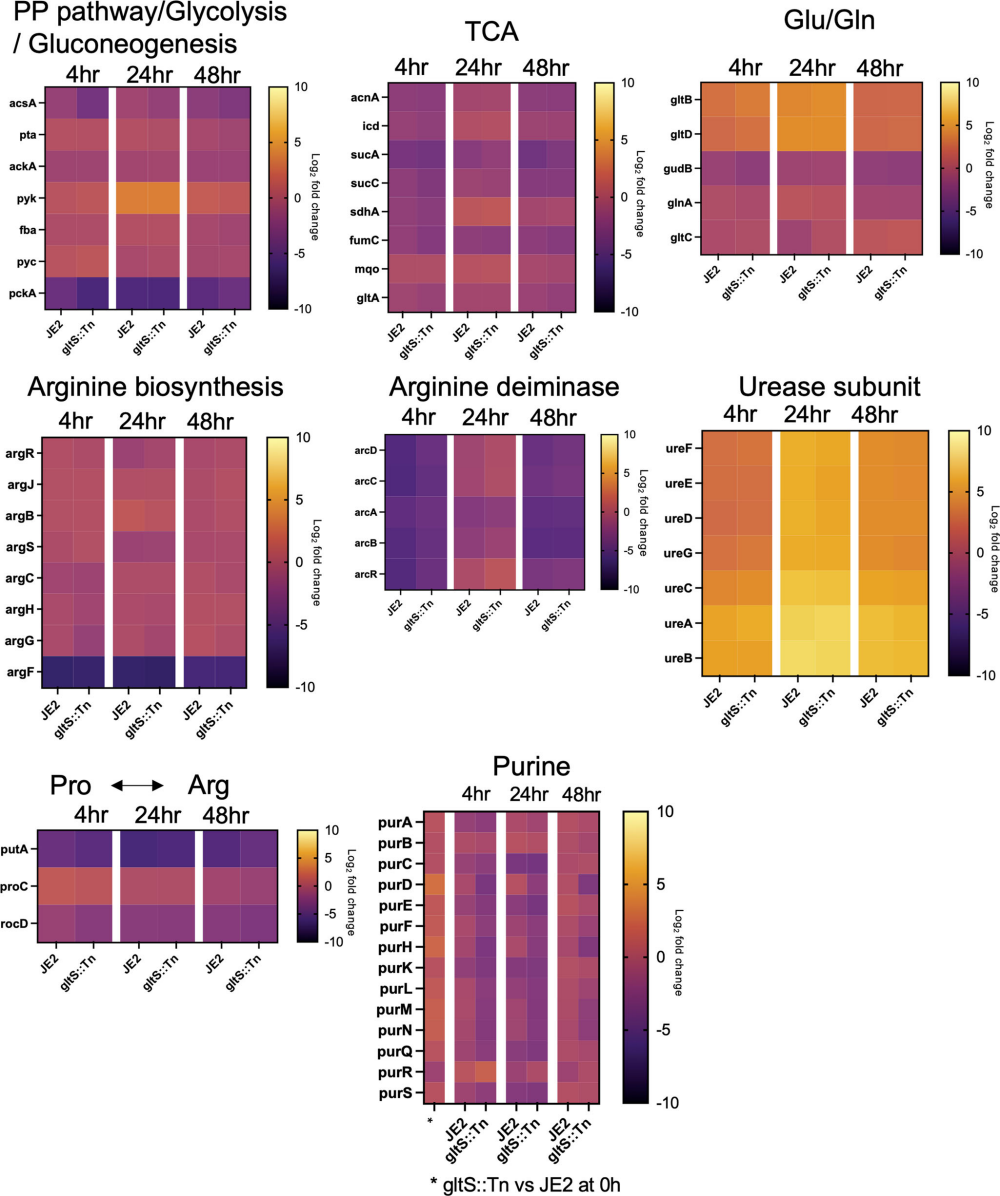
RNA seq analysis of JE2 and *gltS* transposon mutant biofilm cells. Heat map showing the course of change in log_2_ fold change at each time point referenced to the start of biofilm culture (0 h). The bar indicates log_2_ fold change at each time point referenced to the start of biofilm culture (0 h).

First, we examined TCA cycle and urea cycle related genes. The heatmap shows the log_2_ fold change of each gene at indicated time points compared to time point 0 h in each pathway (Fig. 6). TCA cycle-related genes were downregulated in biofilm cells, while arginine production-related genes were upregulated, consistent with our RT-qPCR data. The expression of arginine deiminase^20^, which converts arginine to citrulline and is highly expressed during biofilm growth in multiple bacterial species^21^, peaked at 24 h in both strains, and was higher over time in *gltS::Tn*. Urease is also associated with biofilm formation^22^. The expression of *ureAB* in *gltS::Tn* (0 h) was more than twice that of the parental strain (Supplementary Data 7).

Glutamate production pathway-related genes were also upregulated in the mutant strain. *gltC* positively regulates the expression of GOGAT in *Bacillus subtilis*. Its MRSA homolog in the *gltS* mutant was expressed ~2-fold higher than JE2 at 24 h. Among genes responsible for the pathway that generates glutamate from histidine, *hutI* and *hutU* were found to be upregulated in *gltS::Tn* at time 0 (Supplementary Data 7), which might indicate extra glutamate production pathways in addition to GOGAT.

Interestingly, the expression of *ahpFC*, which serves to protect cells from DNA damage by alkyl hydroperoxides, was higher in the mutant strain at all the four time points (Supplementary Data 7). In addition, at the beginning of biofilm culture or at an earlier time point, presumably before attachment, genes related to reactive oxygen species (ROS), heat shock regulation, biofilm cell attachment, and purine production, such as *dps*, *grpE*, *clpC*, *sdrD* and *purHLMN*, were expressed at higher levels in *gltS::Tn*. These data are consistent with the higher biofilm formation of the *gltS::Tn* strain.

Lastly, the expression of the purine production pathway was higher in *gltS::Tn* only at time point 0 h, and the trend was reversed after 4 h (Fig. 6). Interpretation of this result needs further study.

### Determination of cyclic-di-AMP levels and *dacA* and *gdpP gene expression*

**Fig. 7:**
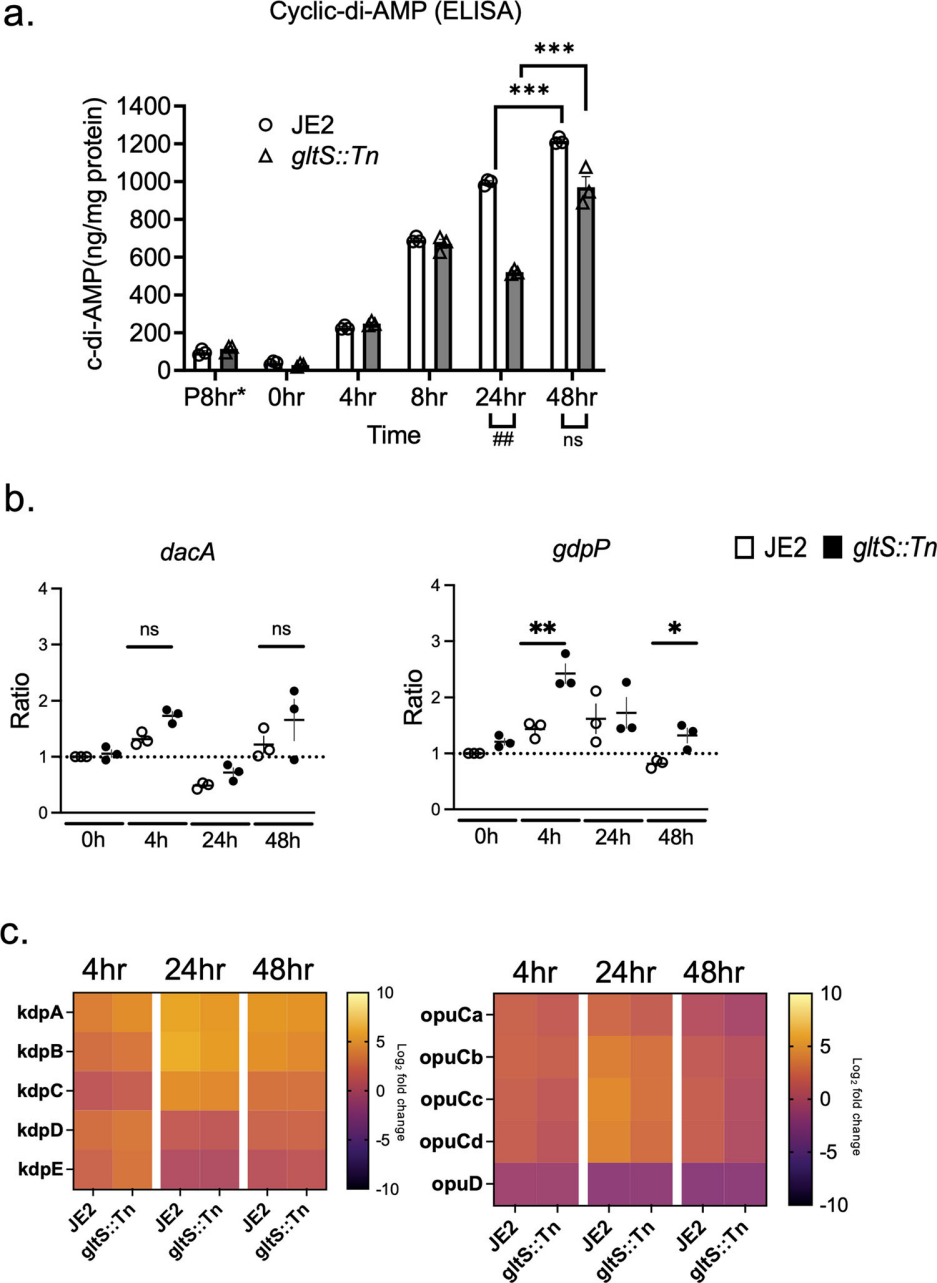
Intracellular c-di-AMP levels and relative expression of genes related to c-di-AMP. **a** Intracellular cyclic-di-AMP levels measured by ELISA. Data are shown as mean ± SD of 3 replicates. Student t-test was used. ***p < 0.001. *P 8 h indicates 8 h after starting planktonic culture. **b** Gene expression ratio of *dacA* and *gdpP* by RT-qPCR. Data are mean ± SD of 4 replicates. Student t-test between strains of samples harvested at the same time. *P < 0.05, **p < 0.01. **c** Heatmap of RNA seq results for *kdp* and *opu* operons. Log_2_ fold change from expression at time 0 for each gene. The bar indicates log_2_ fold change at each time point referenced to the start of biofilm culture (0 h).

Cyclic-di-AMP (c-di-AMP) is an intracellular signaling molecule involved in various functions including ROS production, biofilm formation, and negative regulation of the TCA cycle, particularly in Gram positive bacteria^13,23,24^. Intracellular c-di-AMP levels of the parental strain significantly increased during biofilm formation. At 48 h, it exceeded by 10-fold that of JE2 planktonic cells (Fig. 7a), which correlated with the previous reports describing an association between c-di-AMP and biofilm formation^23,25^. c-di-AMP negatively regulates the TCA cycle, and our gene expression analysis of JE2 indicated the negative regulation of TCA cycle during biofilm formation (Fig. 5). Intracellular c-di-AMP levels of *gltS::Tn* showed a similar trend. However, at 24 h, c-di-AMP level of *gltS::Tn* mutant was reduced by ~50% compared to that of the parental strain. Because glutamine is one of contributors to reduce c-di-AMP levels^13^, higher glutamine in *gltS::Tn*: matches with this c-di-AMP result. This lower c-di-AMP level in *gltS::Tn* is also in line with higher TCA metabolite production in *gltS::Tn*. Interestingly, by 48 h, the c-di-AMP levels in the two strains were comparable. The expression of a c-di-AMP-degrading phosphodiesterase *gdpP* in *gltS::Tn* was approximately twice as high as that of the parental strain (Fig. 7b). *dacA* expression did not show a significant difference between the two strains (Fig. 7b). *gdpP* expression level was higher in *gltS:Tn*, consistent with lower c-di-AMP levels in *gltS* mutant (Fig. 7b).

Glutamate is a major intracellular counter ion for potassium^26^. c-di-AMP binds to K exporters and plays a significant role in osmolarity regulation, mainly by modulating the uptake of potassium and osmolytes^27^. We used RNA seq data of genes involved in potassium channel and osmolytes uptake (*kdp* operon and *opu* operon, respectively) to show the time course of their expression ratios from time 0 in a heatmap (Fig. 7c). Overall, *kdpA-E* expression was upregulated during biofilm formation in both strains. In our screening, *kdpB::Tn* demonstrated enhanced biofilm formation (Fig. 1a). The expression of *opuCa* and *opuD* was downregulated particularly at 48 h when the c-di-AMP level was highest, with the expression of *gltS::Tn* being lower.

### A study using mutants related to arginine synthesis and the urea cycle: the role of citrulline

**Fig. 8:**
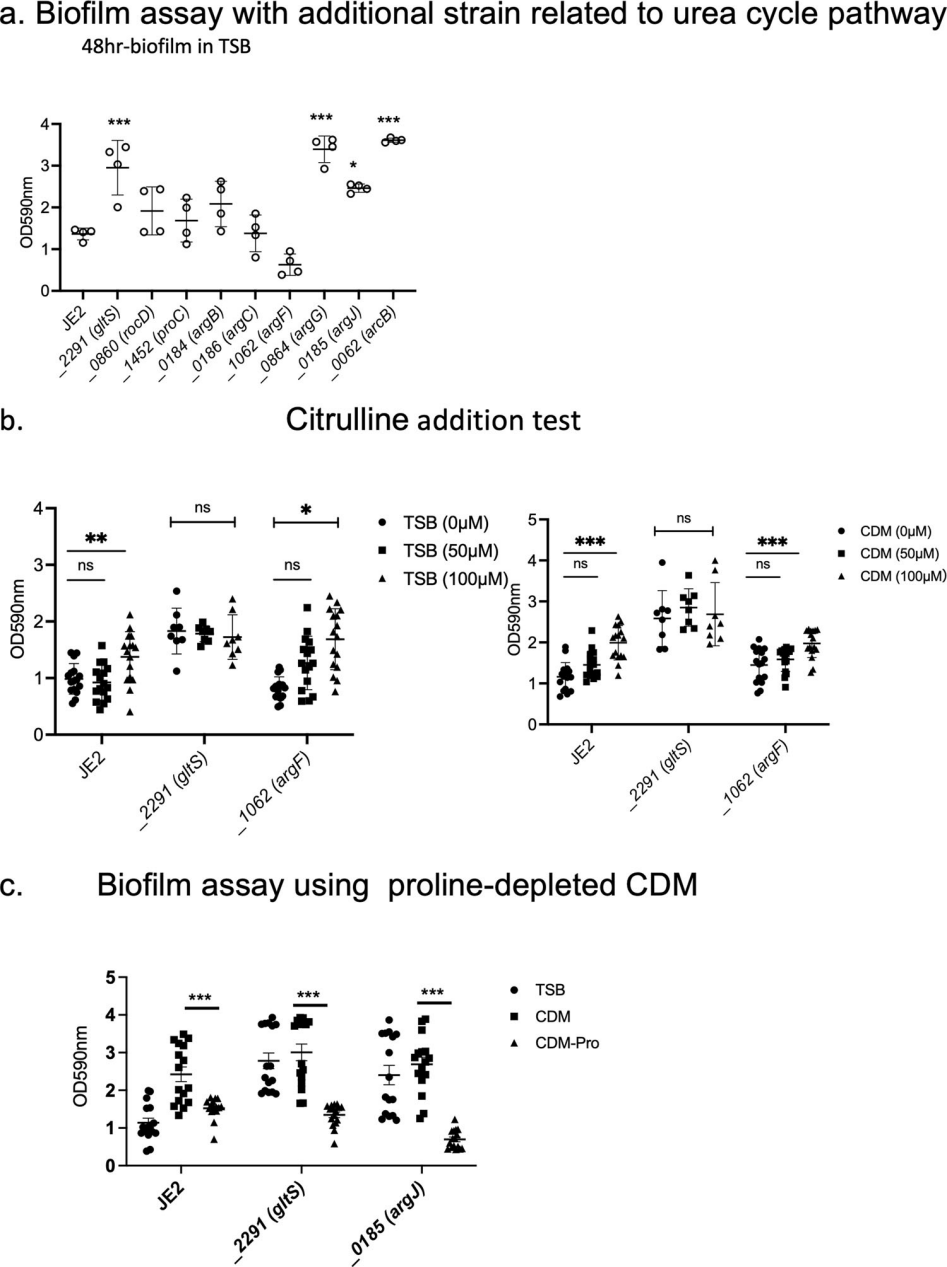
The role of the urea cycle in biofilm formation. **a** Biofilm assay of urea cycle pathway related gene transposon mutants. Biofilm quantification in 1% glucose TSB medium at 48 h. Data are shown as mean ± SD of 8 replicates. One-way ANOVA with *post hoc* Bonferroni‘s multiple comparisons test vs USA300 JE2. ***p < 0.001, *p < 0.05. **b** 48-h biofilm assay using the medium in the presence of additional citrulline (50 µM, 100 µM). Data are shown as mean ± SD of 8 replicates. One-way ANOVA with Bonferroni *post hoc* analysis. *P < 0.05, **P < 0.01, ***P < 0.001. **c** Biofilm assay using proline-depleted CDM. OD value of 48-h biofilm. Data are shown as mean ± SD of 8 replicates. One-way ANOVA with Bonferroni *post hoc* analysis. ***P < 0.001.

With the hypothesis that accelerated urea cycle metabolism would be responsible for enhanced biofilm formation in *gltS::Tn*, we performed a biofilm assay with *Tn* mutants related to arginine/urea metabolism (Fig. 8a). Although not statistically significant, *argF::Tn* markedly reduced biofilm formation, while *argG::Tn*, *argJ::Tn*, and *arcB::Tn* showed significantly enhanced biofilms. No growth difference among the strains was observed (Supplementary Data 2). Because *argF* is an enzyme that mediates the synthesis of citrulline from ornithine, and *argG* and *arcB* mutants enhanced the biofilm, we hypothesized that citrulline would be essential for the biofilm and added citrulline to the TSB culture medium. Both the parental strain and *argF::Tn* showed a significant increase in biofilm formation in a citrulline concentration-dependent manner, although the amount of biofilm formation was not as high as that of *gltS::Tn* (Fig. 8b). Since TSB contains about 50 μM citrulline, we also performed the assay using CDM with or without citrulline. These results were comparable to those of TSB. These results suggest that citrulline would be an important mediator for biofilm formation, and higher citrulline level in *gltS::Tn* (Supplementary Fig. 2b) might have been at least in part responsible for its biofilm phenotype. The mechanism of biofilm enhancement by *argJ::Tn* is unclear. Proline can be a source of citrulline. Thus, we examined the role of proline depletion in biofilm formation. Proline depletion attenuated biofilm formation (Fig. 8c). We also examined the biofilm formation using TCA cycle gene transposon mutants (Supplementary Fig. 5). They did not affect biofilm formation, indicating the involvement of urea cycle in biofilm. Tn mutant of glutamate synthase, *gltD::Tn*, also significantly increased the biofilm formation.

## Discussion

**Fig. 9:**
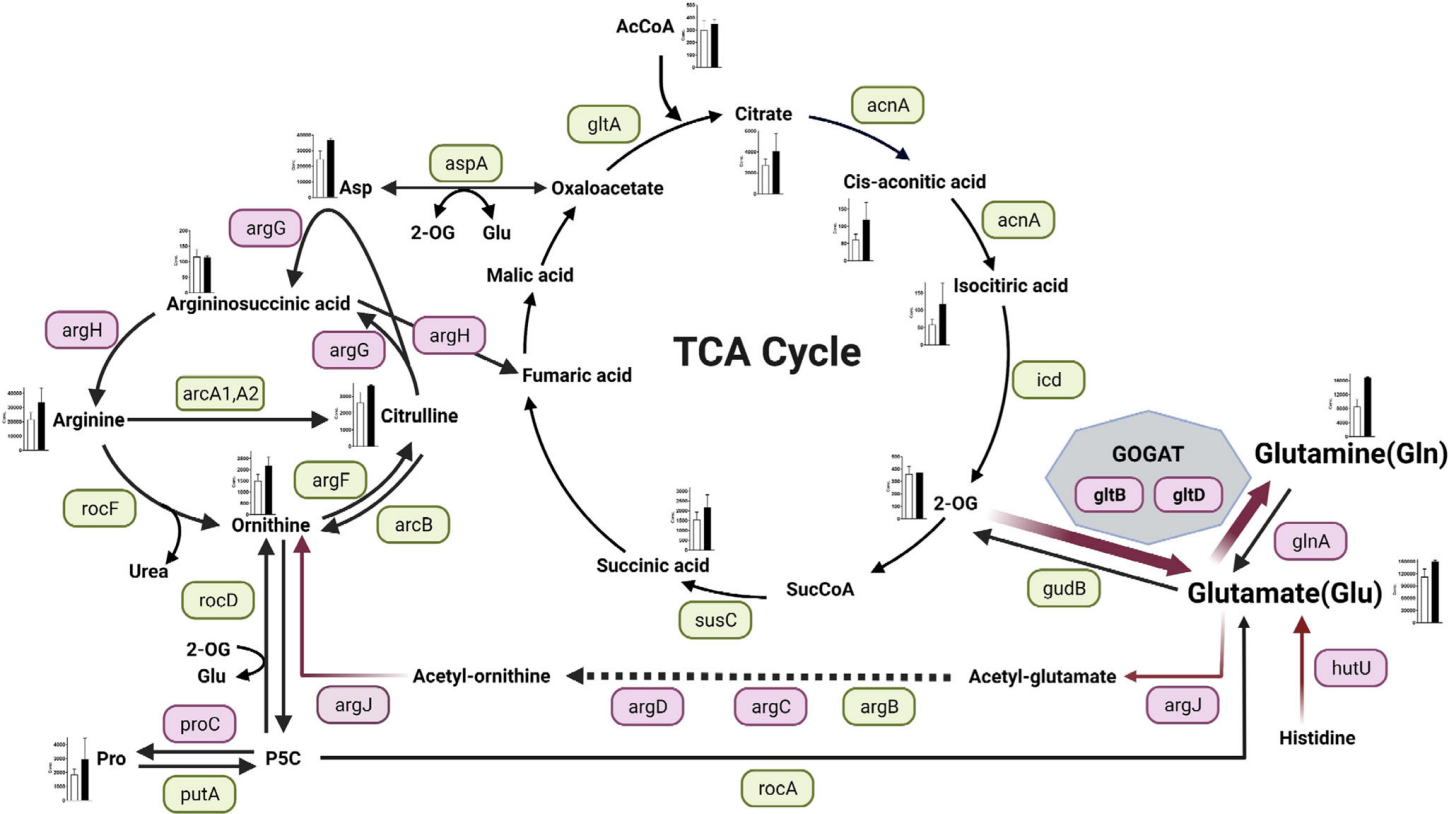
Metabolic changes induced by *gltS::Tn* in TSB. Red indicates that the expression of the gene was higher than that of the parent strain by RT- qPCR at any of the observation times from 0 to 48 h, and green indicates that there was no significant difference between the strains. However, even if there is a significant difference, the expression ratios of both strains that are less than 1-fold of the reference (JE2 time 0) are omitted and shown in green. For hutU, RNAseq data showed that expression was significantly (*p* < 0.05) higher than 2-fold in the *gltS::Tn* strain, hence it is additionally shown in pink. Absolute values of the concentration of each metabolite in the cells of 48-h biofilms are shown in the bar graph. White indicates the parent strain and black indicates *gltS::Tn* strain.

*Staphylococcus aureus* has evolved remarkably to be able to adapt to complex environmental changes in the host, and it forms colonies in a variety of niches^28^. One particularly important example of bacterial adaptation is the ability to grow as part of a sessile community of biofilm. A biofilm is a functional multilayered community of microorganisms, adhering to a surface and organized within a self-produced exopolymeric matrix^29^. Its complex regulation is based on drastic metabolic changes in bacterial cells. In this study, we found that MRSA biofilm formation was enhanced by inactivation of *gltS*. The summary of our findings is shown in Fig. 9.

Glutamate and glutamine serve as the major amino acid donors for all nitrogen-containing compounds in all living organisms, including other amino acids and components of DNA and RNA synthesis. It has been suggested that 80–88% of the nitrogen incorporated into biomass is supplied by glutamate^30^. Therefore, the glutamate pool in cells needs to be kept high^31^. The intracellular glutamine/glutamate ratio serves as an important indicator of nitrogen availability in bacterial cells^32^. In *S. aureus*, there are several pathways to produce glutamate. In *gltS::Tn*, a rapid and intense activation of GOGAT was the primary response to impaired exogenous glutamate uptake for intracellular glutamate pool restoration. The concentrations of both glutamate and glutamine were higher in the *gltS::Tn* strain in the 48 h biofilm. The intracellular glutamate concentration is usually higher than the glutamine concentration because excess glutamine is converted to glutamate via the GOGAT pathway by glutamine oxoglutarate aminotransferase, which is composed of *gltB* and *gltD* protein subunits in *S. aureus*^30^. RNA seq data suggested that histidine to glutamate conversion pathway might also contributed to endogenous glutamate generation in *gltS::Tn*. Furthermore, asparagine serves as an important amino acid for glutamate biosynthesis as shown in Fig. 9, but *aspA* expression level did not differ between the strains, suggesting its limited role of this pathway in endogenous glutamate production by *gltS::Tn*.

The role of GOGAT was extensively studied in *Bacillus subtilis*, a model Gram-positive bacterium. GOGAT (*gltAB*) produces two molecules of glutamate from glutamine and 2-OG^33^. Expression of the *gltAB* gene is triggered by signals originating from carbon and nitrogen metabolism and is dependent on the LysR-type transcriptional activator *gltC* and the global regulator protein of nitrogen metabolism *TnrA*^34^. In the presence of glucose, *gltC* activates transcription of the *gltAB* operon to respond to an increased need for glutamate^35^. On the other hand, in the presence of glutamate, *gltC* is inactive and cannot activate the expression of the *gltAB* operon^30^. It is possible that *gltC* is activated when glutamate uptake is reduced. Although *S. aureus gltC* is present, its function is not known^36^. In a 24-h biofilm, we observed that *gltC* was expressed two-fold higher in *gltS::Tn* compared to the JE2 parent strain. However, the relationship between *gltC* and GOGAT in USA300 remains to be determined.

According to a nuclear magnetic resonance analysis, amino acids other than arginine, histidine, and proline must go through the TCA cycle and GOGAT to produce glutamate^37^. Our data showed increased TCA cycle metabolite levels. TCA cycle receives input at various steps. Metabolite profiles showed that the amount of PP pathway into the TCA cycle was almost the same between the parent and *gltS* mutant. Intermediates from urea cycle can also enter TCA cycle. Argininosuccinic acid and asparagine can enter TCA cycle (Fig. 9). Both *argG* and *argH*, which produce argininosuccinic acid and arginine and fumaric acid from citrulline, showed significantly higher expression in *gltS::Tn* than in the parental strain. Therefore, *gltS::Tn* biofilms may have an increased influx from the urea cycle into the TCA cycle. Compatible with this finding, TCA cycle intermediates such as citrate, cis-aconitic acid, isocitiric acid were detected in higher quantity in *gltS:: Tn* mutant. The fact that the step from isocitiric acid to 2-OG is a rate limiting step likely explains the similar amount of 2-OG between JE2 and *gltS::Tn*.

In *S. aureus*, TCA cycle activity is controlled by several regulators (e.g., *ccpA*, *ccpE*, *codY*, and *rpiRc*) in response to environmental stress or the availability of sugars or amino acids^32^. c-di-AMP is known to be an inhibitory regulator of the TCA cycle in bacteria like *Listeria monocytogenes*^38^. Furthermore, it has recently been identified that the sRNA *RsaE* downregulates mRNAs related to glycine cleavage, TCA cycle, urea cycle, and amino acid metabolism^39^. We observed that the expression of intermediate catalytic gene products was more repressed in biofilm cells than in planktonic cells, although there was no significant difference between the parental and *gltS::Tn* strains in mRNA expression measured by RT-qPCR. The expression ratio of *ccpA* was flat under all conditions that we tested, and thus its influence is difficult to assess. Overall more studies are needed to elucidate the full extent of this complex mechanism.

Experimental evidence indicates that arginine production and glutamate metabolisms are intimately involved in biofilm formation and maturation^40^. Transcriptomic analysis of *S. aureus* planktonic cells and biofilms showed six genes (*rocD*, *gudB*, sucC, *grpE*, *hrcA*, and *clpC*) that were up-regulated during biofilm growth^40^. *rocD* and *gudB* encode ornithine-oxoacid transaminase and glutamate dehydrogenase, respectively, and are involved in the metabolism and biosynthesis of arginine. In MRSA strains, complete inhibition of arginine transport reduced PIA and ammonia accumulation, with negative consequences for pH homeostasis^41^. Arginine has been found to increase stress tolerance and the formation of *S. aureus* persister cells^42^. In the current study, it is plausible that some of the glutamate, following endogenous accelerated production, became arginine via *argJ*. Arginine synthesis from the proline precursor pathway (using *putA* and *proC*) occurs under normal growth conditions, but the arginine pathway through *argJBCD/argGH* plays a role under stress conditions^42^. A direct pathway for the generation of arginine from glutamate via *argJ* has been proposed to degrade arginine in a subsequent arginine deiminase pathway to synthesize ammonia, which can mitigate the cell death-promoting hydroxyl radicals. Arginine is also a substrate for nitric oxide synthase (NOS) in *S. aureus*. Nitric oxide^43^ has been clarified to function in hydrogen peroxide resistance, biofilm formation, and toxin production^44^. Several studies have shown a model in which arginine is preferentially taken up by host innate immune cells and used for inducible nitric oxide synthase (iNOS), arginase, and cell-mediated immunity^45–48^, leading to rapid depletion of arginine in the abscess, effectively reducing the amount of extracellular arginine available in the microenvironment. It has been shown that the expression of both iNOS and arginase is significantly induced during abscess formation by *S. aureus*^49^, further indicating that arginine depletion occurs at the site of infection. Therefore, the enhanced arginine production capacity observed in *gltS::Tn* may be an advantage for the bacteria.

Makhlin et al. have shown that *S. aureus* encodes three arginine biosynthesis regulators, *argR1*, *argR2*, and *AhrC*^50^, which regulate a complex biosynthetic pathway from glutamate and proline. The fact that arginine biosynthesis is not entirely dependent on glutamate is likely to make studies on the regulation of arginine biosynthesis difficult, and further studies are needed to elucidate the details. As *argR2* and *ahrC* function to inhibit arginine biosynthesis via *argG* in the presence of arginine^37^, *argG::Tn* mutant enhanced biofilm formation in our study. Furthermore, *argF::Tn* biofilms was significantly inhibited. The gene is responsible for enzymes required for conversion from or synthesis to citrulline. The importance of citrulline production in biofilm formation was confirmed in our study. However, the full mechanism of how *gltS::Tn* causes higher biofilm formation remains to be determined. This will be further explored in the future.

*S. aureus* synthesizes c-di-AMP from two molecules of ATP by the diadenylate cyclase (DAC) enzyme *dacA*, which is degraded by the phosphodiesterase (PDE) *gdpP*^23^.c-di-AMP was recently discovered and is responsible for many basic cellular functions, mainly within Gram-positive bacteria, including cell wall maintenance, potassium ion homeostasis, glutamate/ glutamine regulation, and DNA damage repair^51,52^. Four types of c-di-AMP receptor proteins have been identified, and these proteins are encoded by *ktrA*, *cpaA*, *kdpD*, and *pstA*^53^. These receptors, in addition to other undiscovered targets, are likely responsible for these functions.

There is a fair body of evidence that c-di-AMP plays a major role in osmotic regulation, primarily by regulating potassium import and export and the uptake of osmotic substances^54–56^. The *kdpDE* two-component system that controls the expression of the high-affinity potassium uptake system in *S. aureus* is regulated by c-di-AMP^55^. Since c-di-AMP positively regulates K efflux, it would be reasonable to expect that its level would be elevated in biofilms under conditions that require osmotic adjustment.

A decrease in c-di-AMP levels leads to an increase in TCA cycle activity and accumulation of intracellular metabolites such as citrate, glutamine and glutamate^38^. According to Zeden et al., the regulation of c-di-AMP synthesis in response to the presence of glutamine, glutamate, or ammonium occurs at the synthetic level, not at the degradative level^13^. However, they did not study biofilms. RT-qPCR results in our current study showed that *gdpP* was significantly upregulated in *gltS::Tn* at 4 h after the start of biofilm incubation and its c-di-AMP levels significantly dropped at 24 h, suggesting the involvement of *gdpP* in the regulation of c-di-AMP levels. However, the level of c-di-AMP for *gltS::Tn* increased again from 24 h to 48 h. Glutamine was higher in *gltS::Tn* at 48 h, but it is possible that other mediators are responsible for the regulation of c-di-AMP at 48-h time points.

The relationship between c-di-AMP and biofilm may not be straightforward as we saw that during the formation of *gltS::Tn* biofilms, c-di-AMP levels once decreased to half the level of the parent strain before increasing again. The stringent response alarmone pentaphosphate ((p)ppGpp) has been known to regulate adaptive responses to starvation conditions, such as amino acid depletion^57^. ppGpp is also involved in the flexible regulation of c-di-AMP to suit the intracellular potassium overload. ppGpp binds to *gdpP* to inhibit the degradation of c-di-AMP. Once degradation is inhibited, synthesis of c-di-AMP resumes, and as long as ppGpp is present, the second messenger is protected from complete degradation^58^.

Furthermore, *gdpP* contributes to eDNA release, which means that when the c-di-AMP level is reduced under biofilm-induced conditions, cell wall integrity is compromised, leading to cell lysis and eDNA release^59,60^. eDNA requires the presence of matrix proteins to attach to cells and acts as an electrostatic net that organizes cells into large clumps^7,61,62^. The eDNA release, triggered by a temporary drop in c-di-AMP levels, may have made the *gltS::Tn* biofilm robust. In fact, the previous study reported that a drop in c-di-AMP triggered biofilm formation^59,63^. c-di-AMP mediated-modulation of glucan producing enzyme *gtfB* expression level is reported to be the mechanism to be responsible for biofilm formation in *Streptococcus mutans*^25^. In the study, higher c-di-AMP levels were associated with biofilm formation but longitudinal change of c-di-AMP was not reported^23^. However, it remains to be determined how and what c-di-AMP affects in the process of biofilm formation in various bacteria including *Staphylococcus aureus*.

Like c-di-GMP, c-di-AMP is importantly involved in biofilm formation in *S. aureus* background strains by other mechanisms^23^. There is a relationship between c-di-AMP and the cell wall properties of Gram-positive bacteria. When c-di-AMP levels are elevated, *S. aureus* can grow in the absence of lipoteichoic acid (LTA). MRSA *gdpP::Tn* mutant strains have an increased level of cross-linked peptidoglycan and are more resistant to antimicrobial agents with cell wall activity^23^. These may be associated with a marked increase in drug resistance in biofilm cells.

Drug that inhibits the synthesis of glutamate have been shown to not only disrupt the structure and biomass of biofilms, but also to inhibit metabolic cooperation between cells in different layers of the biofilm^64^. Our results complement these findings and suggest that intake of exogenous glutamate is able to inhibit biofilm formation with a therapeutic potential.

In conclusion, we found that *gltS* was a critical regulator of biofilm formation by controlling the intake of exogenous glutamate (Supplementary Fig. 6). An intervention to facilitate exogenous glutamate intake by modulating *gltS* may be a potential future therapeutic option to treat biofilm infections.

## Methods

### Bacterial strains and growth conditions

*Staphylococcus aureus* JE2, a plasmid-cured derivative of the community-associated methicillin-resistant USA300 LAC strain, and the defined bursa aurealis JE2 transposon mutants were acquired from the Nebraska transposon mutant library via the Network on Antimicrobial Resistance in *S. aureus* (http://www.narsa.net). The predicted function of genes corresponding to available transposon mutants were examined in Kyoto Encyclopedia of Genes and Genomes (KEGG) database (https://www.genome.jp/kegg/). Ones with predicted ion transporters were chosen for the study. Bacterial strains were grown aerobically overnight at 37 °C and 200 rpm in Tryptic Soy Broth (TSB) containing 0.25% glucose, and when required, erythromycin was added at 10 μg/mL.

### Complementary strain construction

To complement the *gltS* mutant, the *gltS* open reading frame was amplified from JE2 genomic DNA by PCR using primers F5′ – GCGCATATGATAGAACTTAATGCAATTACAAC containing an NdeI site and R5′ – GCGCTCGAGTTAACTAAACCATTGTATGAATCCCA containing an XhoI site. The *gltS* expression plasmid was constructed by cloning the *gltS* gene under control of the *hprK* promoter into the *E. coli* *S. aureus* shuttle vector pOS1^65^. The sequence of *gltS* in pOS1-*gltS* was verified by Sanger sequencing, and the plasmid was amplified in *Escherichia coli* DH5α. The pOS1-*gltS* plasmid was transformed into RN4220 by electroporation and subsequently transduced into JE2 (*gltS::bursa aurealis*) with phage 80α^66^. The complemented strain was selected on TSA containing 10 µg/ml chloramphenicol.

### Chemically defined medium (CDM)

For the biofilm assay and metabolic studies, *S. aureus* was cultured in CDM containing 1% glucose, phosphate buffer, trace minerals, vitamins, nucleobases, nitrogen and sulfur sources and amino acids^67^. The amount of each amino acid was modified to be the same as in TSB. A list of the amounts of amino acids added to the medium is given in Supplementary Data 8. The osmolality of each medium was measured using VAPRO Vapor Pressure Osmometer MODEL 5600 (ELITechGroup, Puteaux, France), and the results are shown in Supplementary Data 8.

### Biofilm assay

We quantified biofilm formation under static conditions using a quantitative crystal violet assay on polystyrene 96-well plates (Corning #3799,Tewksbury, MA) as described previously^68^. In brief, cultures grown overnight in a shaking condition (we called them as planktonic cells) were washed with phosphate-buffered saline (PBS) twice and diluted to an OD at 600 nm of 0.05 in TSB or CDM supplemented with 1% glucose. A total of 150 µL was transferred into the 96 well plates and incubated at 37 °C for 48 h except where specified in the figure legends. After incubation, the supernatants were removed, and the plates thoroughly washed twice with distilled water. We stained with 1% (wt/vol) crystal violet for 15 minutes and washed with distilled water, after which 33% acetic acid was added for quantitative assessment. We determined biofilm biomass using a standard assay measured by OD_590 nm_ using a plate reader. Each sample was tested in eight wells, and each experiment was performed in triplicate.

For the biofilm assay with proteinase K treatment, the supernatant of the 24-h biofilm was aspirated and replaced with a medium containing 2 µg/ml proteinase K (Thermo Fisher Scientific, Waltham, MA). The supernatant of the control samples was replaced with fresh medium. After incubation for 24 h, the biofilm was quantified by crystal violet staining.

We examined the influence of eDNA on biofilm formation by adding DNase I (100 Kunitz/mL; Roche, Mannheim, Germany) at the start time of incubation. Biofilms were quantified after 24 and 48 h. We tested the influence of eDNA for the stability of preformed biofilms by 1-h DNase treatment after 24 and 48 h of biofilm growth. The bacterial culture supernatant on the biofilm was carefully removed. After the wells were dry, we added 200 μL fresh TSB with or without DNase I (100 Kunitz/mL). After 1-h incubation, biofilm biomass was quantified by crystal violet staining as described above.

### Confocal laser scanning microscopy (CLSM) for biofilm samples

JE2 and *gltS::Tn* 48 h biofilms were prepared in two chambered plastic slides (Lab-Tek Permanox Slide, Thermo Fisher Scientific). Biofilms were double stained with the LIVE/DEAD *Bac*Light Bacterial Viability Kit (Molecular Probes, Inc., Eugene, OR). Live cells stained with the green fluorescent dye SYTO 9 (6 µM), and dead cells stained red with propidium iodide (30 µM). Post staining, samples were washed two times with PBS and viewed by CLSM (Zeiss 880 laser scanning confocal with Fast Airyscan). The z-stack images were collected and exported from Zen 3.3 blue edition software and imported into the ImageJ program (USNIH, Bethesda, MD). In some experiments, live staining was performed in 96 well format. Live cells were quantitated by using Synergy plate reader (Bio Tek Instruments; Winooski, Vermont, USA) with the excitation of 485 nm and emission of 538 nm.

### In vivo wound infection mice model

To assess in vivo biofilm formation, we utilized the deep muscle biofilm infection model^69,70^. After anesthesia with ketamine/xylazine, we shaved the hair over the thigh muscle of a mouse and disinfected the surgical area. We made a 1 cm incision with a scalpel directly into the thigh muscle to the depth of the femur, placed one silk suture in the muscle, inoculated *S. aureus* (10^6^ CFU/10 μL) into the incision under the suture, and then closed the skin. The formation of biofilm was observed on the suture by scanning electron microscopy. Sutures removed 48 h after infection were placed in 500 μL of PBS, sonicated for 15 s, serially diluted, plated on TSA plates, and incubated overnight at 37 °C. The number of live bacteria was verified by counting the number of CFU. For muscle samples, each sample was diluted in 1 mL of PBS per 100 gm, and the CFU in the homogenized suspensions was determined by serial dilution and plating. In some experiments, tissues were subjected to IL-6 mRNA RT-qPCR and glucose analysis. Regarding glucose analysis, glucose colorimetric assay kit (Cell Biolabs, Inc. San Diego, CA, USA) was used per the company protocol.

### Scanning electron microscope (SEM) imaging of wound sutures

After removal from the wound, sutures were subjected to fixation using 2.5% glutaraldehyde, 1.25% paraformaldehyde and 0.03% picric acid in 0.1 M sodium cacodylate buffer (pH 7.4). Samples were washed in 0.1 M cacodylate buffer and postfixed with 1% osmium tetroxide (OsO_4_)/1.5% potassium ferrocyanide (KFeCN_6_). Samples were further washed with water and maleate buffer and then incubated in 1% uranyl acetate. The sutures underwent dehydration in grades of alcohol and placed in propylene oxide. The samples were embedded in TAAB Epon (TAAB laboratory equipment Ltd, Berkshire, UK) and polymerized at 60 °C for 48 h. Ultrathin sections (60 nm slice) were cut on a Reichert Ultracut-S microtome, picked up on to copper grids, stained with lead citrate, and examined in a JEOL 1200EX transmission electron microscope or a TecnaiG^2^Spirit BioTWIN. Images were recorded with an AMT 2k CCD camera.

### Histopathology

For histopathological observation, the muscles of infected mice were immersed in 10% formalin for 48 h, and paraffin-embedded sections were sliced, stained with H&E, and visualized by light microscopy.

### Metabolome profiles

Quantitative analysis was performed using capillary electrophoresis mass spectrometry (CE-time-of-flight mass spectrometry (TOFMS) and CE-QqQMS) at Human Metabolome Technologies Inc. (HMT; Yamagata, Japan). *S. aureus* biofilms were incubated as described above, and the biofilm supernatants were gently removed from the wells. The supernatant fluid was also thought to contain bacterial cells, but in order not to regard these as biofilm cells, the sample was centrifuged to remove and sampled as supernatant. The biofilm-associated bacteria were then collected by washing the wells vigorously. Both samples were stored at −80 °C until sample submission. We sent samples to HMT in a frozen condition. A total of 116 metabolites involved in glycolysis, the pentose phosphate pathway, the TCA cycle, the urea cycle, and the metabolism of polyamines, creatine, purine, glutathione, nicotinamide, choline, and amino acids were identified in the HMT metabolite database. The peak area was converted to relative peak area by the following equation.

1 $${{{{{\rm{Relative}}}}}}\; {{{{{\rm{peak}}}}}}\; {{{{{\rm{area}}}}}}=\frac{{{{{{\rm{Metabolite}}}}}}\,{{{{{\rm{peak}}}}}}\,{{{{{\rm{area}}}}}}\,}{{{{{{\rm{Internal}}}}}}\,{{{{{\rm{standard}}}}}}\,{{{{{\rm{peak}}}}}}\,{{{{{\rm{area}}}}}}}$$

The peaks were annotated based on the migration times in CE and m/z values determined by TOFMS. The list of metabolite abbreviations measured is given in Supplementary Data 9.

### RT-qPCR relative gene expression analysis

Total RNA was isolated using a Ribopure Bacteria kit according to the company’s protocol (Life Technologies, Carlsbad, CA). Briefly, bacterial cells were disrupted using Zirconia beads and chloroform was used to layer RNA fraction. A total of 1 μg RNA was purified and first-strand cDNA was synthesized using random primers (Thermo Fisher Scientific). RT-qPCR was performed (StepOnePlus system, Applied Biosystems, Thermo Fisher Scientific Inc., Waltham, MA) with SYBR Green PCR Master Mix (Thermo Fisher Scientific). Annealing temperature of 60 °C with 40 cycles was used. Primers in the study are listed in Supplementary Data 10. For data normalization, housekeeping gene *gyrB* was used as an internal reference, and the fold change in gene expression was calculated using the comparative Ct method (2−ΔΔCt).

When gene × cycle number was Ct and *gyrB* cycle number was C_T_, the mRNA expression level of gene X was calculated as:

2 $$[{{{{{\rm{mRNA}}}}}}\,{{{{{\rm{expression}}}}}}\,{{{{{\rm{level}}}}}}\,{{{{{\rm{of}}}}}}\,{{{{{\rm{gene}}}}}}\,{{{{{\rm{X}}}}}}]={2}^{-({{{{{\rm{Ct}}}}}}-{{{{{\rm{CT}}}}}})}$$

### Measurement of cellular c-di-AMP levels by enzyme-linked immunosorbent assay (ELISA)

We determined intracellular c-di-AMP levels using a cyclic di-AMP ELISA kit (Cayman, Ann Arbor, MI). In brief, cells were cultured in media on a shaker for 8 or 16 h before subjected to biofilm culture (0 h of biofilm). Cells were collected by vigorous aspiration at 4, 8, 24, and 48 h. The bacterial cells were washed three times with PBS (without Ca) and centrifuged to obtain the pellet. Sample of 1 g each cell pellet was lysed with 4 mL of B-PER reagent, the lysates were cleared by centrifugation for 5 min at 16,000 *g* and the supernatant transferred to a new tube. Then the protein concentration was determined using Pierce BCA protein assay kit (Thermo Fisher Scientific). We used this kit to optimize the concentration by applying a serial dilution, and then used an 800-fold dilution. Following the addition of tracer, antibody, and either samples or standards per the company’s protocol, the plate was incubated for 2 h at room temperature and then washed five times with 300 µL of wash buffer. Next, 175 μL of 3,3′,5,5′ tetramethylbenzidine dihydrochloride (TMB) substrate solution was added to each well, and the plate was incubated for 30 min at room temperature. The reaction was then stopped by adding 75 μL of horseradish peroxidase (HRP) stop solution. The absorbance was measured in a plate reader at a wavelength of 450 nm. A calibration curve was constructed from the OD values of the standards at each concentration to identify the level of each sample.

### RNA seq of bacterial genes

Bacteria samples were subjected to RNA purification using RiboPure RNA Purification kit (Thermo Fischer Scientific). The RNA was subjected to bacterial rRNA depletion using Ribo Minus bacteria kit (Thermo Fischer Scientific). RNA samples were quantified using Qubit 2 Fluorometer (Life Technology; Carlsbad, CA), and RNA integrity was checked with Agilent TapeStation (Agilent Technologies; Palo Alto, CA). SMART-Seq v4 Ultra Low Input kit for Sequencing was used for full-length cDNA synthesis and amplification (Clonetech; Mountain View, CA), and Illumina Nextera XT library was used for sequencing the library preparation. Briefly, cDNA was fragmented, and adaptors were added using transposase, followed by limited-cycle PCR to enrich and add index to the cDNA fragments. The final library was assessed with Qubit 2.0 Fluorometer and Agilent TapeStation. The sequencing libraries were multiplexed and clustered on one lane of a flowcell. After clustering, the flowcell was loaded on the Illumina HiSeq instrument according to the manufacturer’s instructions. The samples were sequenced using a 2 × 150 paired end configuration. After investigating the quality of the raw data, sequencing reads were trimmed to remove possible adapter sequences and nucleotides with poor quality using Trimmomatic v.0.36. The trimmed reads were mapped to the USA300_FPR3757 reference genome (https://www.ncbi.nlm.nih.gov/assembly/GCF_000013465.1/) using STAR aligner v.2.5.2b. Only unique reads were counted. After extraction of gene hit counts, the gene hit counts table was used. Using DESeq2, a comparison between the groups of samples was performed. The Wald test was used to generate p-value and Log2 fold changes. Genes with adjusted p-values < 0.05 and absolute log_2_ fold change >1 were called differentially expressed genes. Sequencing data were deposited in Gene Expression Omnibus (GEO)(accession number GSE216751).

### Mass spectrometric analysis of bacteria proteins

Centrifuged cell pellets were fully lysed with 9 M urea buffer in 1× phosphate-buffered saline pH 7.4 with freshly added cOmplete™ Protease Inhibitor Cocktail (Roche) and incubated with rotation at room temperature for 10 min followed by sonication at 4 °C. Supernatants were collected and protein concentrations were measured with Bradford assay (ThermoFisher, Waltham, MA). Extracted proteins were reduced and alkylated with 5 mM Tris(2-carboxyethl) phosphine hydrochloride (TCEP-HCl) (Pierce, Appleton, WI) and iodoacetamide (VWR, Radnor, PA) for 30 min in dark and followed by cysteine blocking for 15 min at a final concentration of 5 mM. Then each lysate was diluted to 1.5 M urea with 1× PBS and digested by trypsin at an enzyme to substrate ratio of 1:50 (w/w, Promega, Madison, WI) for overnight at 37 °C followed by another 2 h of trypsin digestion under the same condition for the next day. Digested peptides were then desalted with home-made C18 Stage-tips^71^. Dried peptides were resuspended in HPLC buffer A (0.1% formic acid in water, v/v) and injected onto a self-packed reversed-phase capillary HPLC column (38 cm length × 100 μm ID, ReproSil-Pur C18, 120 Å pore, 1.9 μm resin, Dr. Maisch GmbH, Ammerbuch, Germany). Mass spectrometry (MS) data were acquired by using Dionex Ultimate^TM^ 3000 RSLCnano (ThermoFisher) concatenated to an Orbitrap Fusion mass spectrometer (ThermoFisher). Peptides were separated at a flow rate of 200 nL/min with a 100 min gradient of 5% to 21% HPLC buffer B (0.1% formic acid in acetonitrile, v/v) in 60 min, to 35% HPLC buffer B in 30 min, and to 90% HPLC buffer B in 10 min. Peptide ions were ionized in a positive polarity mode at a spray voltage of 2.1 kV at an Orbitrap resolution of 120,000 at 200 m/z and a scan range of 380–1580 m/z. Dynamic exclusion was set with an exclusion duration of 30 s and a mass tolerance of ±10 ppm. Internal mass calibration was integrated by EASY-IC and top 12 ions with charge states of 2–6 were fragmented in the linear ion trap by normalized high energy collisional dissociation (HCD) of 35% and an isolation window of 1.6 m/z.

MS data were analyzed by MaxQuant software (version 1.5.3.12)^72^ with the integrated Andromeda search engine against the RefSeq protein database for USA300_FPR3757 (Taxonomy ID 451515) concatenated with common contaminant database from Maxquant. Database searching results was filtered at the 1% false discovery rate (FDR) using the target-decoy strategy for both peptide and protein identifications. Trypsin was selected as the proteolytic enzyme with a maximum of 2 missing cleavages. Cysteine carbamidomethylation was set as a fixed modification and methionine oxidation, protein N-termini acetylation were set as variable modifications. Label-free quantification with iBAQ analysis was enabled. Mass spectrometry data was deposited in Proteomics Identification Database (PRIDE, accession number: PXD37860).

### Statistics and reproducibility

Data were analyzed as indicated in the corresponding figure legends. All the statistical calculations other than metabolome profiles and RNA sequencing were performed using PRISM 9 software (GraphPad Software; La Jolla, CA, USA). Statistical significance was defined as *p* < 0.05. Biofilm assays and RT-qPCR were performed at least in triplicate. All data is presented as mean ± standard error of the mean (SEM) or mean ± standard deviation (SD). We used student *t* test, and one-way ANOVA with Bonferroni *post hoc* analysis.

### Reporting summary

Further information on research design is available in the Nature Portfolio Reporting Summary linked to this article.

## Supplementary information

Supplementary Information

Description of additional supplementary data

Supplementary Data 1

Supplementary Data 2

Supplementary Data 3

Supplementary Data 4

Supplementary Data 5

Supplementary Data 6

Supplementary Data 7

Supplementary Data 8

Supplementary Data 9

Supplementary Data 10

Supplementary Data 11

reporting summary

## Data Availability

All the relevant data were included in the manuscript. Main figure raw data are included in Supplementary Data 11. RNA sequencing data was deposited in Gene Expression Omnibus (GEO)(accession number GSE216751) and proteomics data was deposited in Proteomics Identification Database (PRIDE) (accession number PXD037860). Analysis of all the metabolites we measured. Bar indicates z-score with z-score row normalization.
