# Supplementary material for: GltS regulates biofilm formation in methicillin-resistant Staphylococcus aureus
Source: Commun Biol. 2022 Nov 23;5:1284. doi: 10.1038/s42003-022-04239-2 (PMC9684512; doi:10.1038/s42003-022-04239-2)
Supplement: Supplementary file 1 — Supplementary Information [file 42003_2022_4239_MOESM1_ESM.pdf]

Supplemental Information

GltS* regulates biofilm formation in methicillin-resistant *Staphylococcus aureus

Miho Shibamura-Fujiogi[1,2], Xiaogang Wang[3], Wiriya Maisat[1,2], Sophia Koutsogiannaki[1,2], Yunan Li[4], Yue Chen[4], Jean C. Lee[3], Koichi Yuki[1,2]

[1]Department of Anesthesiology, Critical Care and Pain Medicine, Cardiac Anesthesia Division, Boston Children's Hospital, Boston, MA, USA

[2]Department of Anaesthesia and Immunology, Harvard Medical School, Boston, MA, USA

[3]Department of Medicine, Brigham and Women's Hospital and Harvard Medical School, Boston, MA, USA

[4]Department of Biochemistry, Molecular Biology and Biophysics, University of Minnesota, Minneapolis, MN, USA

Supplementary Figures

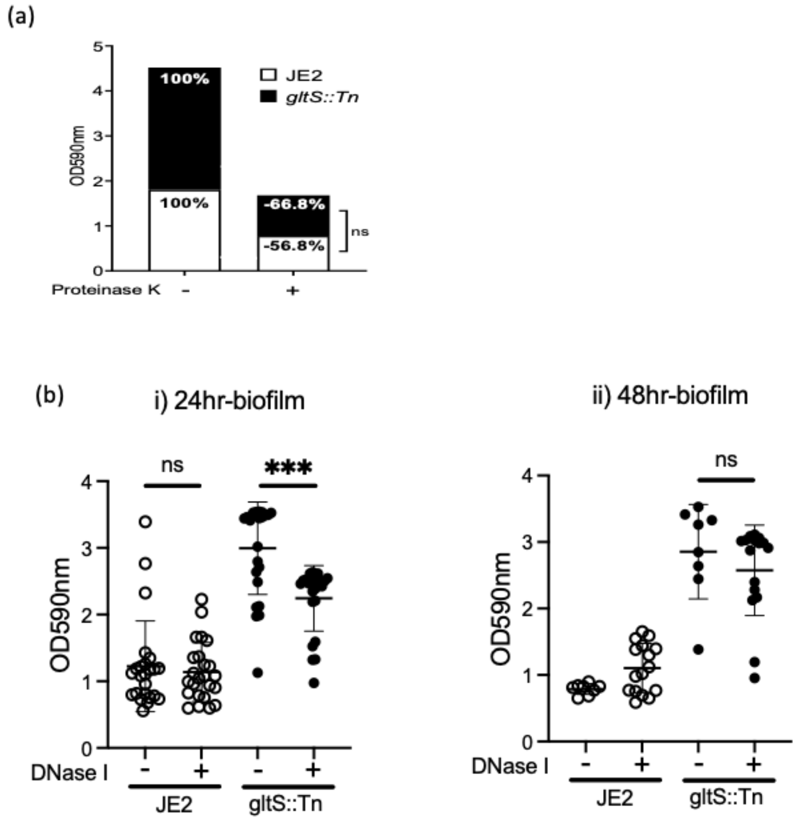

Supplementary Figure 1. The contribution of protein and DNA to MRSA biofilm formation

a) Proteinase K treatment of biofilm samples. After the supernatant of the biofilm was removed at 24 h, the cells were incubated with medium containing 2 µg/mL proteinase K for another 24 h before the biofilm assay was performed. Compared to the control, the biofilm was reduced by 56.8% for JE2 and 66.8% for *gltS::Tn*. The statistical analysis was done using the Student t test; no statistical significance was noted (p=0.532).

b) Biofilm assay with DNase I treatment. 100 knitz DNase I was added after 24 or 48 h, and incubated at 37°C for 1 h before assay. Only the 24-hour biofilm showed a significant volume loss after the treatment. Data showed mean +/- S.D. of 8-16 samples. Student t-test was used for statistical analysis. ***p<0.001.

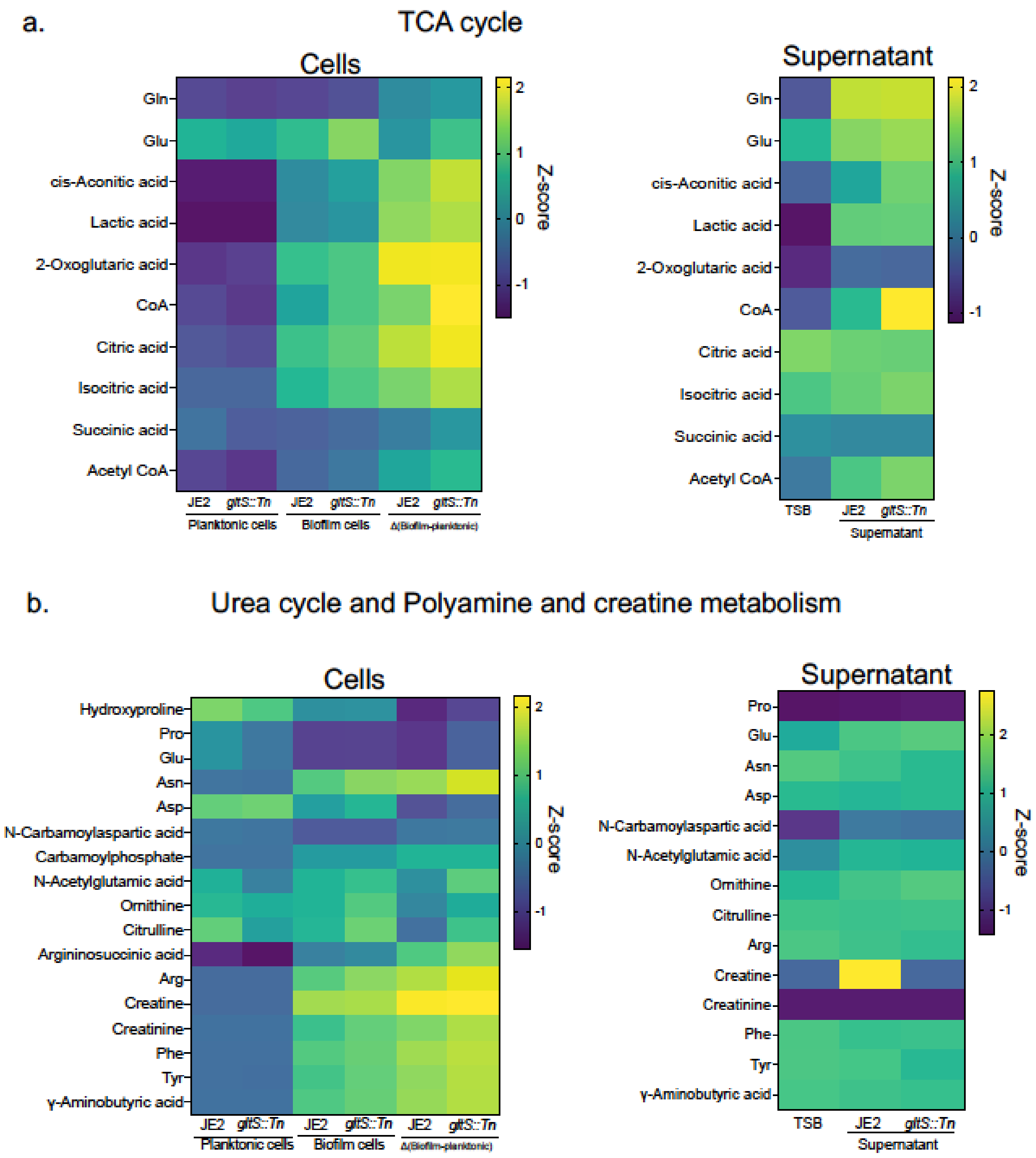

Supplementary Figure 2. Heatmap of metabolome profiles of planktonic and biofilm cells and culture supernatants for (a) TCA cycle and (b) Urea cycle.
Bar indicates z-score values after z-score row normalization.

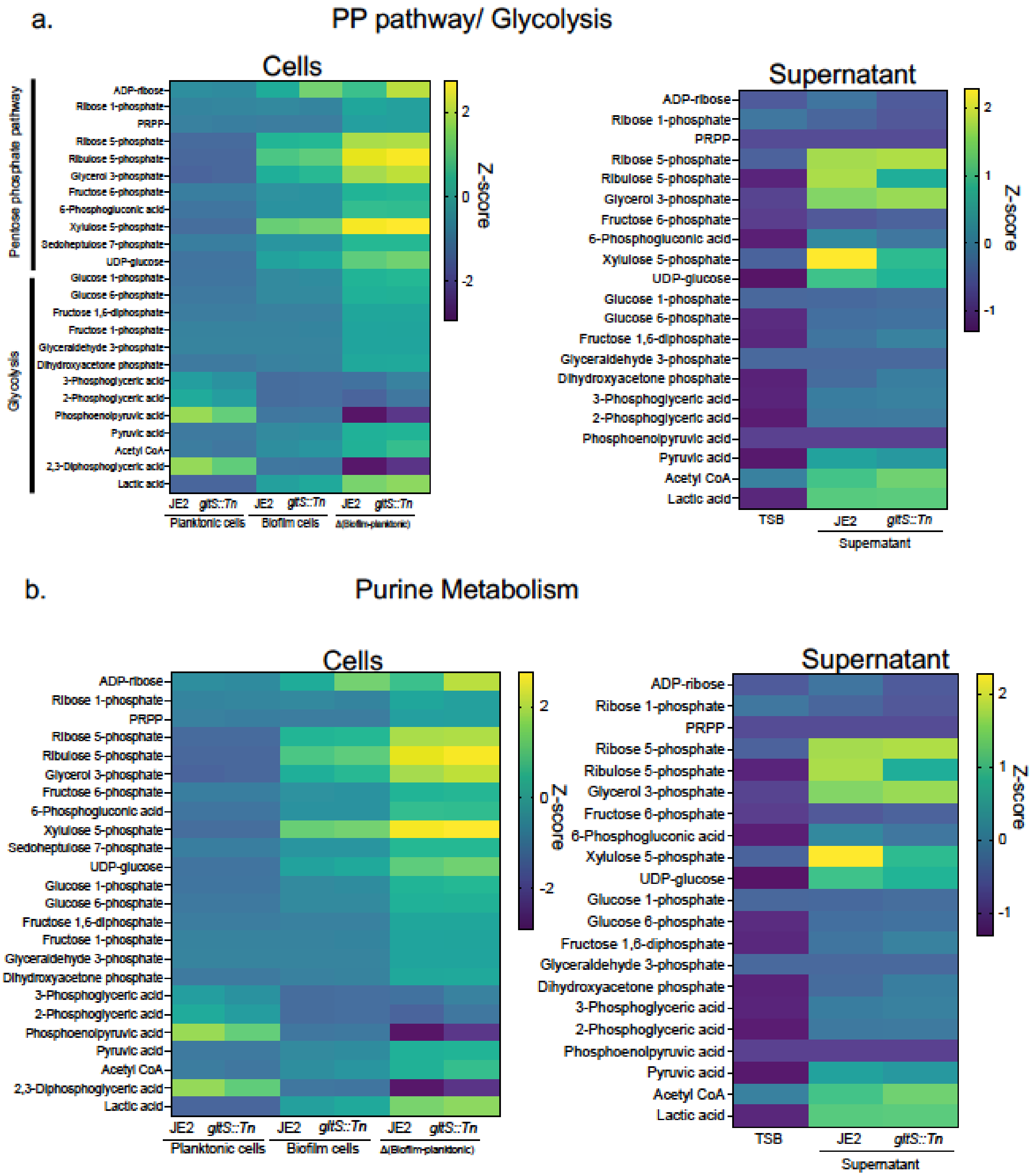

Supplementary Figure 3. Heatmap of metabolome profiles of planktonic and biofilm cells and culture supernatants for (a) PP pathway/glycolysis and (b) Purine metabolism
Bar indicates z-score values after z-score row normalization.

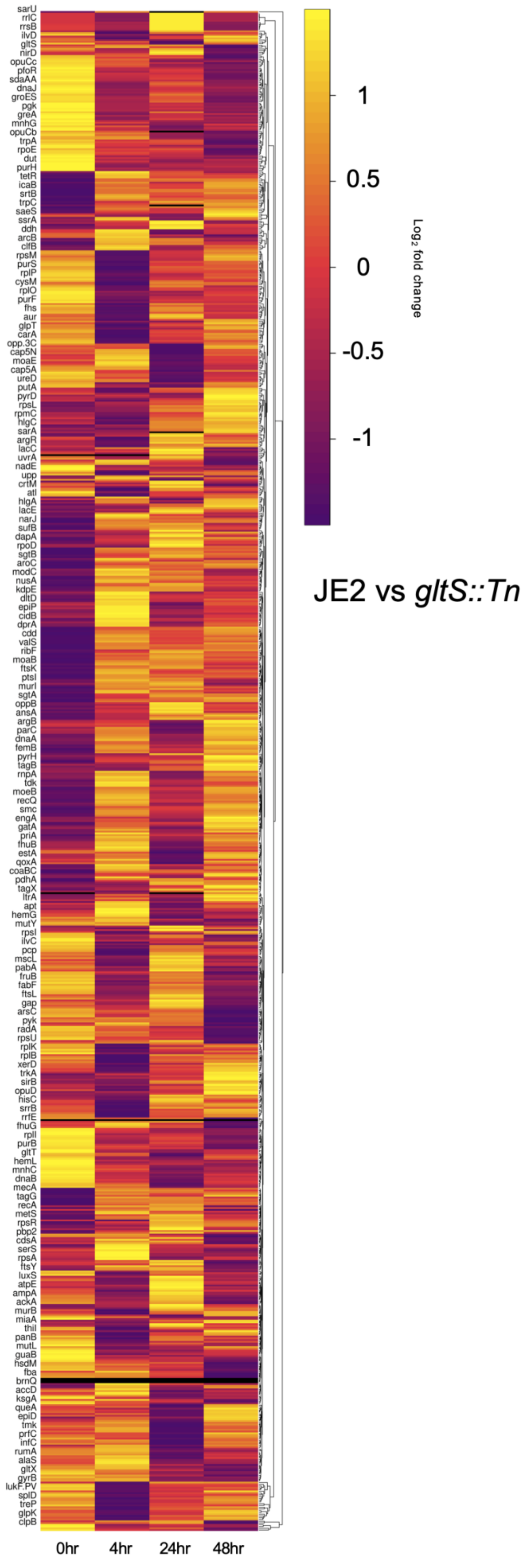
sarU
rrlC
rrsB
ilvD
gltS
nirD
opuCc
pfoR
sdaAA
dnaJ
groES
pgk
greA
mnhG
opuCb
trpA
rpoE
dut
purH
tetR
icaB
srtB
trpC
saeS
ssrA
ddh
arcB
clfB
rpsM
purS
rplP
cysM
rplO
purF
fhs
aur
glpT
carA
opp.3C
cap5N
moaE
cap5A
ureD
putA
pyrD
rpsL
rpmC
hlgC
sarA
argR
lacC
uvrA
nadE
upp
crtM
atl
hlgA
lacE
narJ
sufB
dapA
rpoD
sgtB
aroC
modC
nusA
kdpE
dltD
epiP
cidB
dprA
cdd
valS
ribF
moaB
ftsK
ptsI
murI
sgtA
oppB
ansA
argB
parC
dnaA
femB
pyrH
tagB
rnpA
tdk
moeB
recQ
smc
engA
gatA
priA
fhuB
estA
qoxA
coaBC
pdhA
tagX
ltrA
apt
hemG
mutY
rpsI
ilvC
pcp
mscL
pabA
fruB
fabF
ftsL
gap
arsC
pyk
radA
rpsU
rplK
rplB
xerD
trkA
sirB
opuD
hisC
srrB
rrfE
fhuG
rplI
purB
gltT
hemL
mnhC
dnaB
mecA
tagG
recA
metS
rpsR
pbp2
cdsA
serS
rpsA
ftsY
luxS
atpE
ampA
ackA
murB
miaA
thiI
panB
mutL
guaB
hsdM
fba
brnQ
accD
ksgA
queA
epiD
tmk
prfC
infC
rumA
alaS
gltX
gyrB
lukF.PV
splD
treP
glpK
clpB
0hr
4hr
24hr
48hr
1
0.5
0
-0.5
-1
Log₂ fold change
JE2 vs gltS::Tn

Supplementary Figure 4. RNA seq experiment of JE2 and *gltS::Tn* strains

Heat map of 864 differentially expressed genes (DEGs), *gltS::Tn* vs JE2 log_2 fold change at each time point. Bar indicates log_2 fold change at each time point referenced to the start of biofilm culture (0 hour). Purple represents downregulated genes, and yellow represents upregulated genes. Each column represents one dataset, and each row represents one gene. The heat map was generated by using Heatmapper (http://www.heatmapper.ca/). The list of up- or down-regulated genes at 0 h, 4 h, 24 h, and 48 h is shown in Supplementary data 7.

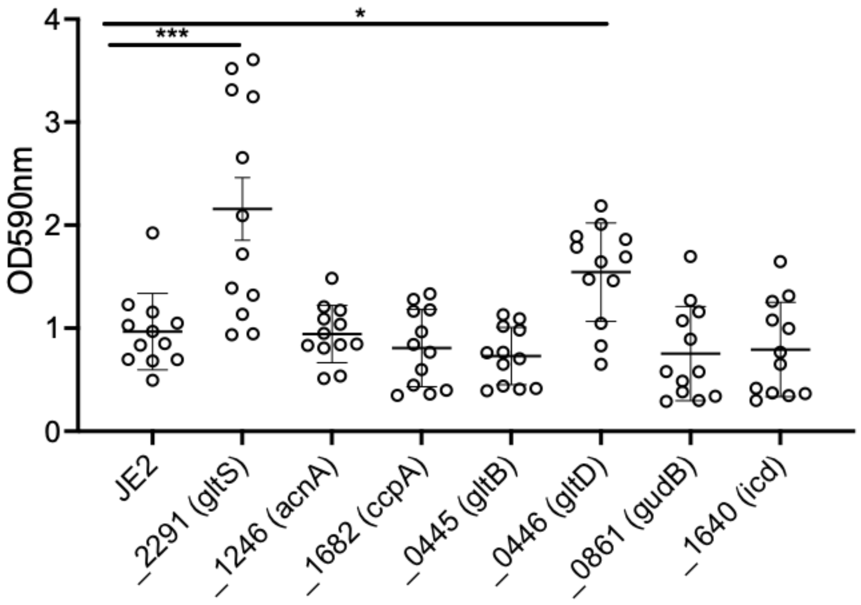

Supplementary Figure 5. Biofilm tests using Tn mutants of enzyme genes involved in TCA cycle and glutamate metabolism.

Biofilm assay of TCA cycle and glutamate metabolism related gene transposon mutants. Biofilm quantification in 1% glucose TSB medium at 48 h. Data are shown as mean +/- S.D. of 16 replicates. One-way ANOVA with *post hoc* Bonferroni multiple comparisons test vs USA300 JE2. ***$p<0.001$, * $p<0.05$.

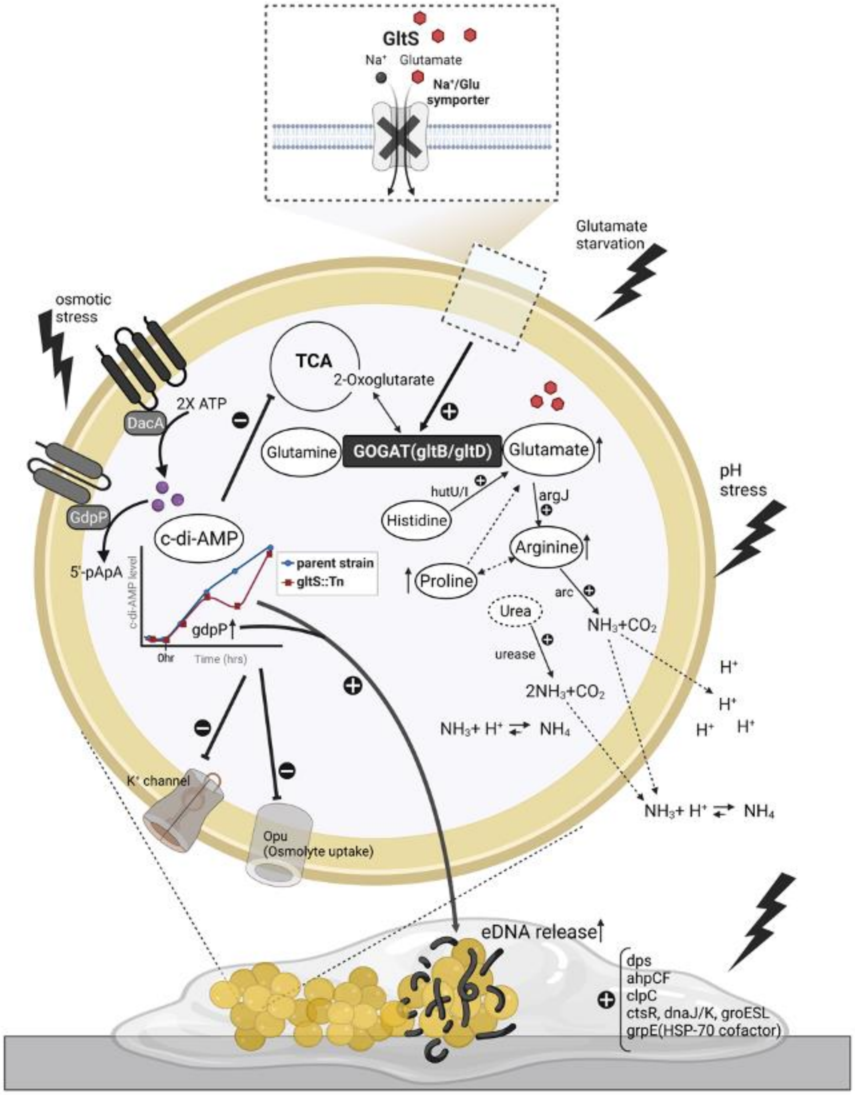

Supplementary Figure 6. The summary of this study.
The figure was created by Miho Shibamura-Fujiogi with the software by BioRender.com.

Supplementary References

1. Frees, D., *et al.* Clp ATPases are required for stress tolerance, intracellular replication and biofilm formation in Staphylococcus aureus. *Mol Microbiol* **54**, 1445-1462 (2004).
2. Chastanet, A., Fert, J. & Msadek, T. Comparative genomics reveal novel heat shock regulatory mechanisms in Staphylococcus aureus and other Gram-positive bacteria. *Mol Microbiol* **47**, 1061-1073 (2003).
3. Mashruwala, A.A. & Boyd, J.M. The Staphylococcus aureus SrrAB Regulatory System Modulates Hydrogen Peroxide Resistance Factors, Which Imparts Protection to Aconitase during Aerobic Growth. *PloS one* **12**, e0170283 (2017).
4. Xu, C.-G., *et al.* Comparative Proteomic Analysis Provides insight into the Key Proteins as Possible Targets Involved in Aspirin Inhibiting Biofilm Formation of Staphylococcus xylosus. *Frontiers in Pharmacology* **8**(2017).
5. Stapleton, M.R., *et al.* Characterization of IsaA and SceD, two putative lytic transglycosylases of Staphylococcus aureus. *J Bacteriol* **189**, 7316-7325 (2007).
6. Yang, N.J., *et al.* Antibody-mediated neutralization of perfringolysin o for intracellular protein delivery. *Mol Pharm* **12**, 1992-2000 (2015).
7. Makhlin, J., *et al.* Staphylococcus aureus ArcR controls expression of the arginine deiminase operon. *J Bacteriol* **189**, 5976-5986 (2007).
8. Askarian, F., *et al.* The interaction between Staphylococcus aureus SdrD and desmoglein 1 is important for adhesion to host cells. *Sci Rep* **6**, 22134 (2016).
9. Daly, K.M., *et al.* Production of the Bsa Lantibiotic by Community-Acquired <i>Staphylococcus aureus</i> Strains. *J Bacteriol* **192**, 1131-1142 (2010).
10. Morollo, A.A. & Bauerle, R. Characterization of composite aminodeoxyisochorismate synthase and aminodeoxyisochorismate lyase activities of anthranilate synthase. *Proceedings of the National Academy of Sciences of the United States of America* **90**, 9983-9987 (1993).
11. Manna, A.C. & Cheung, A.L. sarU, a sarA homolog, is repressed by SarT and regulates virulence genes in Staphylococcus aureus. *Infection and immunity* **71**, 343-353 (2003).
12. Wang, G., *et al.* Novel Inhibitor Discovery of Staphylococcus aureus Sortase B and the Mechanism Confirmation via Molecular Modeling. *Molecules* **23**(2018).
13. Reed, S.B., *et al.* Molecular characterization of a novel Staphylococcus aureus serine protease operon. *Infection and immunity* **69**, 1521-1527 (2001).
