# Supplementary material for: GltS regulates biofilm formation in methicillin-resistant Staphylococcus aureus
Source: Commun Biol. 2022 Nov 23;5:1284. doi: 10.1038/s42003-022-04239-2 (PMC9684512; doi:10.1038/s42003-022-04239-2)
Supplement: Supplementary file 2 — Description of additional supplementary data [file 42003_2022_4239_MOESM2_ESM.pdf]

Description of Additional Supplementary Data

Supplementary Data 1. (a) List of ion transporter mutants in USA300, (b) Additional mutants tested

Supplementary Data 2. Growth curve of JE2 and all the mutants tested. Growth curve was obtained by measuring O.D. of all the strains at 1, 2, 4, 6, 8, 10, 12 and 24 hours. Data were shown as mean +/- S.D.

Supplementary Data 3. Metabolome data. Data of all the metabolites measured for (a) biofilm cells and (b) supernatant.

Supplementary Data 4. Metabolic parameters for biofilm cells.

Supplementary Data 5. Metabolic pathway analysis.

Supplementary Data 6. Mass spectrometry analysis of TCA cycle related enzyme levels.

Supplementary Data 7. Genes with at least 2-fold increased or decreased mRNA levels in *gltS::Tn* cells grown in TBS medium compared to JE2.

Supplementary Data 8. Properties of the culture medium (a) Osmolarity. (b) Amount of amino acid.

Supplementary Data 9. Abbreviated names.

Supplementary Data 10. Primers used in this study.

Supplementary Data 11. Raw data set in the main figures.
